# USP7 promotes temozolomide resistance by stabilizing MGMT in glioblastoma

**DOI:** 10.1038/s41419-025-07969-3

**Published:** 2025-08-20

**Authors:** Jiabing Li, Xiaorong Feng, Zhaohui Liu, Yunfang Deng, Zhiming Sun, Bei Chen, Lihui Wu, Xiaolong Wang, Lin Miao, Liyuan Zeng, Lei Hu, Yuming He, Ying Sheng, Yue Liu, Yu Zhao

**Affiliations:** 1 The National & Local Joint Engineering Laboratory of Animal Peptide Drug Development, College of Life Sciences, Hunan Normal University, Changsha, Hunan 410081 China; 2 Peptide and Small Molecule Drug R&D Platform, Furong Laboratory, Hunan Normal University, Changsha, Hunan 410081 China; 3 Institute of Interdisciplinary Studies, Hunan Normal University, Changsha, Hunan 410081 China

**Keywords:** Enzyme mechanisms, Cancer therapeutic resistance, Ubiquitylation

## Abstract

Glioblastoma (GBM), a World Health Organization (WHO) grade IV glioma, is one of the most lethal brain tumors, with a poor prognosis and limited treatment options. Temozolomide (TMZ), a first-line chemotherapeutic agent, often proves ineffective due to resistance and toxicity associated with overexpressed *O*^6^-methylguanine-DNA-methyltransferase (MGMT). In this study, we identified ubiquitin-specific protease 7 (USP7) as a nuclear regulator of MGMT stability and TMZ resistance. USP7 binds directly to MGMT via its UBL domain, counteracts K48-linked ubiquitin chains, and prevents MGMT proteasomal degradation. This functional relationship is further supported by their nuclear colocalization. Strikingly, this study, together with previous findings, establishes USP7 as a key integrator of all three major alkylation repair pathways through its role in stabilizing alkylation repair proteins. USP7 stabilizes MGMT through a dual mechanism, thereby modulating the direct reversal repair pathway. Inhibition or knockdown of USP7 reduces MGMT levels, as well as those of XPC, ALKBH2, and ALKBH3, impairs DNA repair capacity, and sensitizes GBM cells to TMZ, enabling effective treatment with reduced TMZ dosages. Clinically, tissue microarray analyses reveal that USP7 and MGMT co-overexpression in GBM correlates with poor patient survival. Collectively, our results uncover a new and direct role for USP7 in MGMT-mediated direct reversal repair and TMZ resistance, positioning USP7 as a distinctive integrator of alkylation repair pathways. Targeting USP7 provides mechanistic insights into regulating diverse alkylation repair pathways and offers a strategy to enhance the efficacy of combination chemotherapies, including TMZ and other alkylating agents, by modulating distinct repair mechanisms in GBM.

## Introduction

Glioblastoma (GBM), a WHO grade IV glioma, is one of the deadliest brain tumors with a poor prognosis and limited treatment options. Standard treatments for GBM include surgery, radiotherapy, and chemotherapy. Surgical resection improves the median survival by approximately three months, and postoperative radiotherapy enhances it to about eight months. Temozolomide (TMZ) addition further improves survival, though the median survival remains about fifteen months [1, 2]. TMZ is a potent DNA alkylating agent that methylates nucleotide positions, including the *O*^6^ position of guanine, leading to DNA damage and inducing tumor cell apoptosis [3]. Although TMZ shows significant efficacy against GBM, resistance frequently develops. TMZ treatment can also cause substantial dose-dependent toxicities, such as bone marrow suppression and gastrointestinal complications, which severely impair the quality of life of patients [4–6]. Understanding the molecular mechanisms underlying TMZ resistance and devising strategies to reduce its dosage while maintaining efficacy is critical for improving GBM treatment.

Cells utilize multiple mechanisms to preserve genomic integrity in response to continuous threats from both endogenous and exogenous damaging agents. DNA alkylation is a major form of nucleic acid damage, and repairing alkylated bases is critical because these lesions are mutagenic and may cause DNA breaks. Alkylation damage is repaired primarily by three major pathways: MGMT-mediated direct reversal, demethylation by specific demethylases, and base excision repair (BER). MGMT directly repairs mutagenic and cytotoxic *O*^6^-alkylguanines, which, if left unrepaired, can cause apoptosis and genotoxicity [1, 2]. Although alkylation-based chemotherapy is widely used in cancer treatment, its clinical efficacy and toxicity vary markedly owing to diverse DNA lesions, highlighting the necessity for coordinated alkylation repair to limit toxicity [7]. MGMT is overexpressed in approximately 80% of human brain tumors and is strongly associated with TMZ resistance, particularly in GBM [8]. In GBM, MGMT overexpression counteracts TMZ-induced DNA damage at the alkylated *O*^6^ position of guanine, promoting cell survival, proliferation, and resistance to TMZ. Therefore, elucidating the mechanisms underlying MGMT upregulation is essential to overcome TMZ resistance in GBM.

Although the transcriptional regulation of MGMT by various transcription factors (e.g., HIF-1α, p53, NF-*κ*B, and AP-1) has been extensively investigated [9, 10], accumulating evidence underscores the critical role of post-translational modifications (PTMs) in modulating MGMT activity [11–19]. Among these PTMs, ubiquitination has been demonstrated to be a critical modification of MGMT. Its stability is tightly regulated by ubiquitination and deubiquitination. While ubiquitination by E3 ligases such as RAD18, E6AP, and TRIM72 promotes MGMT degradation [15, 16, 19], the deubiquitinase (DUB) USP19 has recently been identified as a regulator of MGMT stability [17]. Although USP19 is predominantly cytoplasmic, MGMT is primarily nuclear [19–21]. The role of nuclear DUBs in MGMT regulation and TMZ resistance in GBM remains an important unanswered question.

DUBs play an important role in maintaining protein homeostasis through deubiquitination [22]. Among the DUB family, the nuclear-enriched enzyme USP7 is particularly important due to its diverse roles in cellular processes, including oncogenesis (through stabilization of oncoproteins) and the DNA damage response (DDR), where it regulates key repair proteins, such as p53 and Chk1 [23–32]. USP7 has recently been shown to participate in alkylation damage repair. In particular, USP7 promotes base excision repair (BER) by stabilizing repair proteins, such as CSB and XPC [33–36]. USP7 also facilitates demethylation by interacting with OTUD4 to stabilize demethylases, as demonstrated in our previous study [37]. Thus, USP7 modulates two of the three major alkylation repair pathways: BER and demethylation. However, the role of USP7 in direct reversal, an important mechanism in TMZ resistance, remains unexplored.

In this study, we show that USP7, a nuclear DUB previously identified as important for indirectly facilitating demethylation, plays a new and direct role in promoting MGMT-mediated direct reversal repair, positioning USP7 as a key integrator of alkylation repair pathways. By stabilizing MGMT through a distinct dual mechanism, USP7 supports alkylation damage repair and contributes to TMZ resistance. Inhibition or genetic deletion of USP7 disrupts multiple alkylation damage repair pathways, raising the possibility that USP7 could be targeted to enhance the efficacy of combination chemotherapy, including TMZ and other alkylating agents, by modulating distinct repair mechanisms in GBM.

## Materials and methods

### Cell culture

HEK293T and T98G (glioblastoma) cells, obtained from the American Type Culture Collection (ATCC), were cultured in DMEM (10313039, Gibco, NY, USA) supplemented with 10% fetal bovine serum (FBS, 10099-141 C, Gibco, USA) at 37 °C in a humidified 5% CO_2_ atmosphere. All cell lines were confirmed negative for mycoplasma contamination using the Myco-Lumi™ Mycoplasma Detection Kit (C0297, Beyotime, China).

### Antibodies

Antibodies were purchased from the following companies: The antibodies against USP7 (26948-1-AP), MGMT (17195-1-AP), His (66005-1-Ig), and HA (51064-2-AP) were obtained from Proteintech (China), as were the HRP-conjugated anti-rabbit (SA00001-2) and anti-mouse (SA00001-1) IgG antibodies. The anti-Flag antibody was obtained from Sigma (F3165, MO, USA). HRP-conjugated β-actin (A00730, Genscript, China) and β-tubulin (E021043, Earthox, USA) were used as loading controls.

### Plasmid constructs, transfection, and lentiviral infection

Full-length and truncated forms of USP7 constructs were generated by PCR and cloned into appropriate vectors. For shRNA-resistant USP7 (WT) and USP7 (C223S) constructs, eight silent mutations were introduced into the ORF sequence targeted by shUSP7. Transient transfections were performed using Lipo8000™ Transfection Reagent (C0533, Beyotime, China) according to the manufacturer’s instructions. Lentiviruses were generated by transfecting HEK293T cells with pLKO.1 or pHAGE and packaging vectors. The supernatants were collected 72 h post-transfection and filtered through 0.45 μm filters. Cells were infected with lentiviruses in the presence of 6 μg/mL polybrene for 12 h. After replacing the medium, cells were selected using blasticidin or puromycin. shRNA knockdown efficiency was validated by western blotting.

### Immunoprecipitation and western blotting

Immunoprecipitation was performed as previously described [37]. In brief, HEK293T cells were transfected with the indicated plasmids using Lipo8000™ Transfection Reagent for 2–3 days. Cells were harvested and resuspended in lysis buffer (150 mM NaCl, 50 mM Tris-HCl, pH 7.9, 1% Triton X-100, 10% glycerol, 2 mM DTT, supplemented with protease and phosphatase inhibitors). The lysates were clarified and incubated with anti-Flag M2 affinity beads (A2220, Sigma, USA) or HA-agarose beads (SC-7392AC, Santa Cruz, USA) at 4 °C overnight. For denatured immunoprecipitation (denatured IP), the lysates were boiled in TBS containing 1% SDS and then diluted with lysis buffer to 0.1% SDS before immunoprecipitation. Bound proteins were eluted with Laemmli buffer and analyzed by western blotting.

### Protein expression and purification

Recombinant His-tagged USP7 protein was purified from Rosetta (DE3) *Escherichia coli* using Ni-NTA agarose beads (P2241, Beyotime, China). Briefly, bacterial cultures were grown to an OD_600_ of 0.8. Isopropyl β-D-1-thiogalactopyranoside (IPTG, 7 mM) was added to induce protein expression at 16 °C overnight. Cells were pelleted by centrifugation (8000 × *g*, 15 min, 4 °C) and resuspended in lysis buffer. The lysates were incubated with Ni-NTA agarose beads overnight at 4 °C. After washing with washing buffer (150 mM NaCl, 50 mM Tris-HCl, pH 7.9, 1% Triton X-100, 10% glycerol, 2 mM DTT) containing 20 mM imidazole, His-tagged USP7 was eluted using lysis buffer containing 400 mM imidazole.

### Immunopurification and silver staining

T98G cells stably expressing Flag-MGMT were lysed and immunoprecipitated using anti-Flag M2 affinity beads (Sigma) overnight at 4 °C on a rotating wheel. The immunoprecipitates were washed five times with washing buffer, and the bound proteins were eluted using Flag peptides (F3290, Sigma, USA). The eluates were collected, separated by SDS-PAGE, and visualized by silver staining using a Silver Staining Kit (C500021, Sangon Biotech, China).

### Immunofluorescence assay

Cells were seeded onto circular coverslides in 12-well plates, treated with 200 μM temozolomide for different time points, and fixed with 4% paraformaldehyde for 30 min. Cells were permeabilized with 0.5% Triton X-100 in PBS for 20 min at room temperature. After three washes with TBST, cells were incubated with primary antibodies overnight at 4 °C. The next day, the coverslips were washed four times with TBST and incubated with Hoechst and secondary antibodies for 1 h at 37 °C. After washing, cells were visualized using a fluorescence microscope (Olympus, Japan). The Pearson correlation coefficient (PCC) was used to assess the correlation between USP7 and MGMT. PCC values were calculated from 34 cells pooled from two biologically independent experiments (n = 34). The sample size was determined based on common practice in image-based co-localization studies. No formal power analysis was performed.

### Cycloheximide chase assay

For the cycloheximide (CHX) chase assay, cells were treated with 180 μg/mL CHX for different durations. Cells were harvested and subjected to western blotting to assess protein stability. Band intensities from western blotting were quantified using ImageJ. Quantification was based on three biologically independent experiments (n = 3), which is a standard sample size commonly used for biochemical assays. No formal power analysis was performed.

### RNA extraction and qRT-PCR

Total RNA was extracted using the RNAeasy™ RNA Isolation Kit (R0026, Beyotime, China), according to the manufacturer’s protocol. RNA concentration and purity were assessed using a NanoDrop spectrophotometer, measuring absorbance at 260 nm and calculating the A260/280 and A260/230 ratios. Complementary DNA (cDNA) was synthesized using BeyoFast™ SYBR Green qPCR Mix (D7260, Beyotime, China) following the manufacturer’s instructions. β-actin was used as the internal control. The primers used for qRT-PCR are listed as follows: MGMT, forward primer, 5’-TTT TCC AGC AAG AGT CGT TCA C-3’, and reverse primer, 5’-GGG ACA GGA TTG CCT CTC AT-3’; β-actin, forward primer, 5’-TCA TCA CTA TTG GCA ACG AGC GGT TC-3’, and reverse primer, 5’-TAC CAC CAG ACA GCA CTG TGT TGG CA-3’.

### Mass spectrometry analysis

Potential MGMT-interacting proteins were co-immunoprecipitated from T98G cells and analyzed by LC-MS/MS, as described previously [37, 38]. In brief, T98G cells stably expressing Flag-MGMT were lysed, and the lysates were incubated with anti-Flag M2 affinity beads overnight at 4 °C with gentle agitation. Immunoprecipitates were washed five times with washing buffer, and the bound proteins were eluted using Flag peptides, followed by mass spectrometry analysis.

### In vitro deubiquitination assay

HEK293T cells co-transfected with Flag-MGMT and HA-Ub plasmids were treated with MG132 (a proteasome inhibitor) and then lysed in a lysis buffer. Poly-HA-ubiquitinated Flag-MGMT was isolated from cell extracts using anti-Flag M2 beads and eluted using Flag peptides. Recombinant USP7 or its catalytically inactive mutant (C223S), expressed and purified from *Escherichia coli*, was incubated with poly-HA-ubiquitinated Flag-MGMT in deubiquitination buffer (50 mM Tris-HCl, pH 7.9, 5% glycerol, 150 mM NaCl, 2 mM DTT) for the indicated time points at 37 °C. The resulting reactions were terminated by adding SDS loading buffer and boiling, followed by analysis by western blotting using an anti-HA antibody.

### Affinity purification of K48-linked polyubiquitinated proteins using Halo-TUBE

K48-linked ubiquitin chains and ubiquitinated proteins were isolated from cell extracts using a tandem ubiquitin-binding entity (TUBE) containing Halo-tag (Halo-TUBE) resin under denatured conditions. Briefly, after TMZ or P22077 treatment, cells were harvested, lysed, and denatured by boiling in TBS buffer containing 1% SDS, and then diluted with lysis buffer to reduce the SDS concentration to 0.1% prior to affinity purification. Extracts were incubated with 10 μL of HaloLink™ resin (G1915, Promega) pre-bound to purified Halo-TUBE protein (10 μg per sample). The clarified extracts were incubated with Halo- TUBE-bound resin overnight at 4˚C with rotation. Subsequently, the resin was washed five times with washing buffer, boiled in Laemmli sample buffer, and analyzed by western blotting.

### Cell viability assay

To assess cell viability in response to temozolomide (TMZ), cells were seeded in 96-well plates and treated with TMZ or vehicle control for 48 h the following day. Cell viability was assessed using the Cell Counting Kit-8 (CCK-8, Beyotime, China) following the manufacturer’s instructions.

### Colony formation assays

To assess clonogenic survival, T98G cells stably expressing shRNAs targeting USP7 or MGMT were seeded in 24-well plates (400 cells per well) and incubated overnight to allow for cell attachment. The following day, DMSO (vehicle control) or TMZ was added to each well at the indicated concentrations, and the medium was replaced every three days with fresh TMZ. After 9–10 days, the medium was removed, and the cells were fixed with 4% paraformaldehyde (P0099, Beyotime) for 10 min at room temperature, followed by staining with 0.1% crystal violet (C0121, Beyotime) according to the manufacturer’s instructions. The plates were washed three times with distilled water and air-dried, and the number of colonies containing more than 50 cells was counted under a microscope.

### Tissue microarray

A glioma tissue microarray (TMA, Cat. No. N092Ct01) was purchased from Zhongke Guanghua (Xi’an, China) Intelligent Biotechnology Co., Ltd. The TMA comprised 92 glioma samples of varying histological grades with paired adjacent normal tissues. Immunofluorescent staining for MGMT (1:500 dilution) and USP7 (1:500 dilution) was performed using TSA Plus Fluorescence Kits (Servicebio) according to the manufacturer’s instructions. The TMA slides were scanned using the Pannoramic SCAN system (3DHISTECH), and the resulting images were analyzed using ImageJ software. The immunofluorescence (IF) score was calculated by multiplying the percentage of positive cells score (1 = 0–20%; 2 = 21–50%; 3 = 51–80%; 4 = 81–100%) by the staining intensity score (1 = weak, 2 = moderate, 3 = strong). An IF score ≤4 was classified as low expression in the analyzed samples, and a score > 4 was classified as high expression.

### Quantification and statistical analysis

Graphs were generated and statistical analyses were performed using GraphPad Prism 7.0. Results are presented as mean ± standard deviation (SD) from at least three independent experiments. The correlation between nuclear USP7 and MGMT signals in immunofluorescence images was assessed using the Pearson correlation coefficient (PCC), which is appropriate for evaluating the linear association between two continuous, approximately normally distributed variables. The assumptions of linearity and bivariate normality were visually assessed. No samples or cells were excluded from the analysis. Randomization was not applicable to the in vitro experimental design. Blinding was not performed. *p* values < 0.05 were considered statistically significant (* *p* < 0.05, ** *p* < 0.01, *** *p* < 0.001).

## Results

### USP7 binds directly to MGMT

**Fig. 1:**
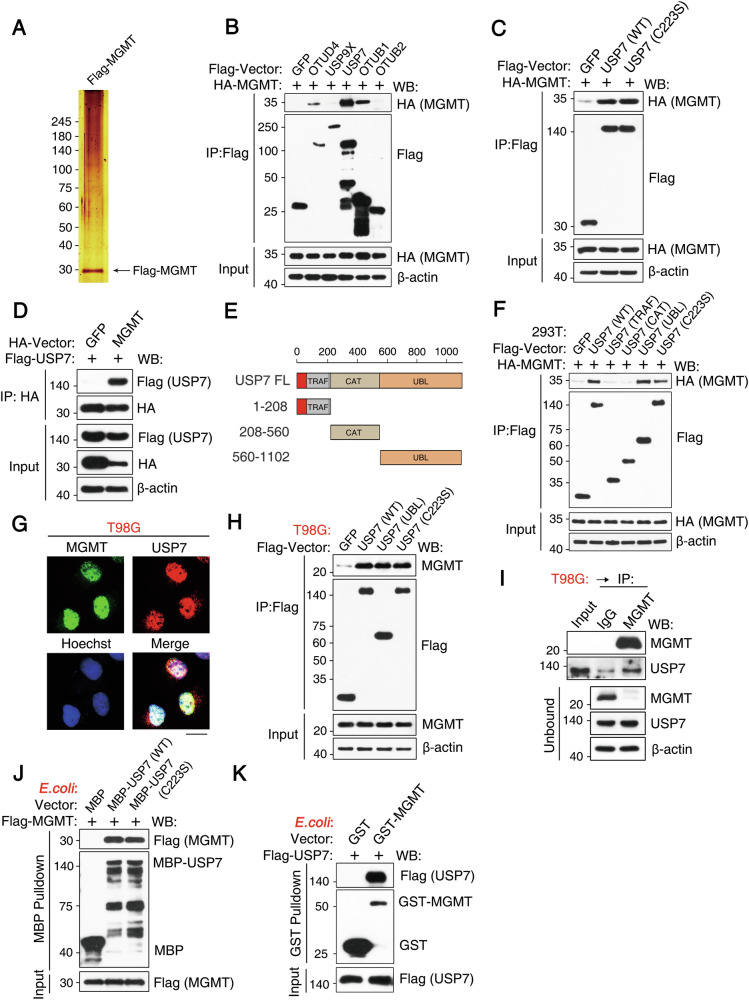
USP7 directly interacts with MGMT. **A** The Flag-MGMT complex was purified from T98G cells using anti-Flag antibody-conjugated M2 beads, followed by SDS-PAGE analysis and silver staining. **B** HEK293T cells stably expressing HA-MGMT were transfected with various Flag-tagged DUBs, followed by immunoprecipitation and western blotting. **C** HEK293T cells stably expressing HA-MGMT were transfected with Flag-USP7 WT or Flag-USP7 C223S, followed by immunoprecipitation and western blotting with the indicated antibodies. **D** HA immunoprecipitation was performed after the expression of the indicated vectors, followed by western blotting. **E** A schematic representation of the Flag-tagged full-length USP7 deletion mutants is provided. **F** HEK293T cells stably expressing HA-MGMT were transfected with Flag-tagged USP7 or its truncated mutants, followed by immunoprecipitation and western blotting. **G** Diffuse nuclear co-localization of MGMT (Green) and USP7 (Red) in T98G cells (Hoechst Blue nuclei) by confocal immunofluorescence. Scale bar: 20 µm. Pearson Correlation Coefficient (PCC) analysis confirmed strong positive nuclear co-localization (mean = 0.67 ± 0.20 SD; n = 34 cells, 2 independent experiments). **H** Whole-cell lysates from T98G cells transduced with the indicated USP7 constructs were subjected to immunoprecipitation, followed by western blotting. **I** Endogenous USP7 was immunoprecipitated with an anti-MGMT antibody from T98G cells to assess the interaction. **J**, **K** In vitro, MBP and GST pulldown assays demonstrate the direct interaction between USP7 and MGMT.

To investigate the regulation of MGMT, a key enzyme in the direct reversal pathway of DNA alkylation repair, Flag-tagged MGMT was expressed in T98G cells (a glioblastoma cell line). Interacting protein complexes were immunoprecipitated using anti-Flag affinity resin. LC-MS/MS analysis identified USP7 and USP31 as potential MGMT-interacting proteins (Fig. 1A; Supplementary Table S1). USP7 is known to stabilize substrates by removing K48-linked ubiquitin chains, while USP31 has been shown to counteract K63-linked ubiquitination [39–41]. These findings suggest a role for USP7 in regulating MGMT and thus, potentially influencing the direct reversal repair pathway. Notably, USP7 also plays an indirect role in the demethylation pathway by interacting with OTUD4 to stabilize demethylases, as we previously demonstrated [37].

To further investigate the interaction between USP7 and MGMT, we performed co-immunoprecipitation (co-IP) assays following the co-expression of Flag-tagged DUBs with HA-tagged MGMT in HEK293T cells. USP7, OTUD4, and OTUB1 co-immunoprecipitated with MGMT, while USP9X and OTUB2 did not (Fig. 1B). The USP7-MGMT interaction was independent of USP7 catalytic activity (Fig. 1C). Reciprocal co-IP assays provided further evidence for this interaction (Fig. 1D). We generated USP7 deletion mutants to map the MGMT-binding domain(s), and these mutants were analyzed by co-IP (Fig. 1E). Notably, the UBL domain of USP7 (residues 560-1102) was identified as being critical for MGMT binding (Fig. 1F).

Immunofluorescence staining demonstrated nuclear colocalization of MGMT and USP7 in T98G cells, confirmed by quantitative analysis (Fig. 1G). Analysis of endogenous MGMT and USP7 protein levels in T98G cells further supported their in vivo interaction (Fig. 1H, I). Direct binding of MBP-tagged USP7 and Flag-tagged MGMT, both expressed and purified from bacteria, was confirmed using a pull-down assay (Fig. 1J) and corroborated by reciprocal GST pulldown assays using GST-MGMT (Fig. 1K). Collectively, these results demonstrate that USP7 directly interacts with MGMT in the nucleus.

### USP7 regulates K48-linked ubiquitination of MGMT

**Fig. 2:**
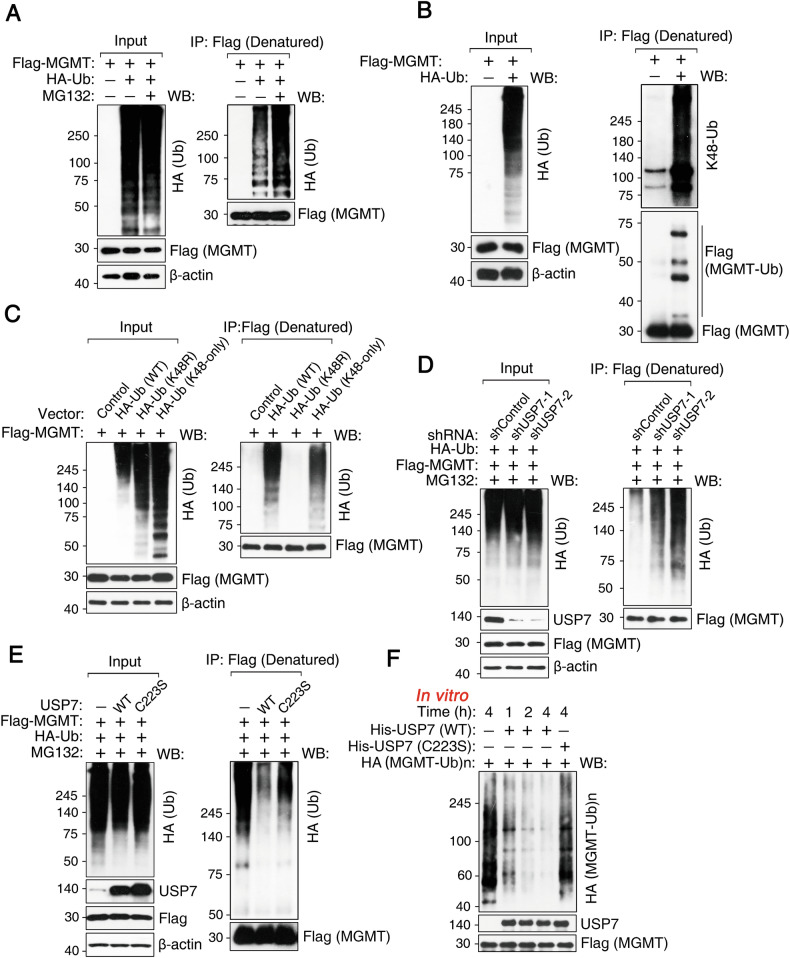
USP7 regulates K48-linked ubiquitination of MGMT. **A** HEK293T cells stably expressing Flag-MGMT were transfected with HA-Ub and treated with MG132, a proteasome inhibitor, followed by Flag immunoprecipitation under denatured conditions (denatured IP) and western blotting. **B** Cellular lysates from panel **A** were analyzed by western blotting with an anti-Ub-K48 antibody. **C** HEK293T cells expressing Flag-MGMT were transfected with HA-Ub WT or its mutant constructs, followed by immunoprecipitation and western blotting. **D** HEK293T cells expressing Flag-MGMT were transduced with control or two independent shRNAs targeting USP7, then treated with MG132 (5 µM) overnight, followed by immunoprecipitation and western blotting. **E** HEK293T cells stably expressing Flag-MGMT were transfected with USP7 WT or USP7 C223S and treated with MG132 (5 µM) overnight, followed by immunoprecipitation and western blotting. **F** In vitro deubiquitination assays were performed by incubating ubiquitinated MGMT proteins with purified USP7 or USP7 C223S at 37 °C for the indicated time points, followed by western blotting.

While MGMT ubiquitination is known to be critical for its regulation [17], the precise type of ubiquitin linkage and the functional implications of this modification remain to be fully elucidated. We then characterized the type of MGMT ubiquitination in HEK293T cells following co-transfection with Flag-MGMT and HA-ubiquitin constructs by denatured immunoprecipitation (IP) and western blotting analysis. Treatment with the proteasome inhibitor MG132 resulted in an accumulation of ubiquitinated MGMT conjugates (Fig. 2A), suggesting a role for proteasomal degradation in regulating MGMT levels. Denatured IP experiments confirmed the covalent attachment of ubiquitin to MGMT. K48-linked ubiquitin chains on MGMT were detected using a K48 linkage-specific antibody following denatured IP and subsequent western blotting analysis (Fig. 2B). To validate K48 linkage specificity, similar experiments were carried out using the K48R and K48-only ubiquitin mutants. These results showed that MGMT ubiquitination was almost completely abolished when using the K48R ubiquitin mutant (in which the K48 lysine residue is mutated to arginine), whereas MGMT ubiquitination was still detectable using the K48-only mutant (in which all lysine residues except K48 are mutated to arginine) (Fig. 2C). These results indicated that MGMT is modified by K48-linked ubiquitination, consistent with a role in proteasomal degradation.

USP7 knockdown increased K48-linked MGMT ubiquitination (Fig. 2D), whereas overexpression of wild-type, but not catalytically inactive (C223S), USP7 reduced it (Fig. 2E), indicating that USP7 catalytic activity is required for the deubiquitination of MGMT. For in vitro deubiquitination assays, purified ubiquitinated MGMT conjugates were incubated with recombinant USP7. Wild-type (WT) USP7, but not the C223S mutant, efficiently removed ubiquitin from the MGMT conjugates in a time-dependent manner (Fig. 2F). These in vivo and in vitro data demonstrate that USP7 deubiquitinates MGMT by removing K48-linked ubiquitin chains.

### USP7 regulates MGMT stability

**Fig. 3:**
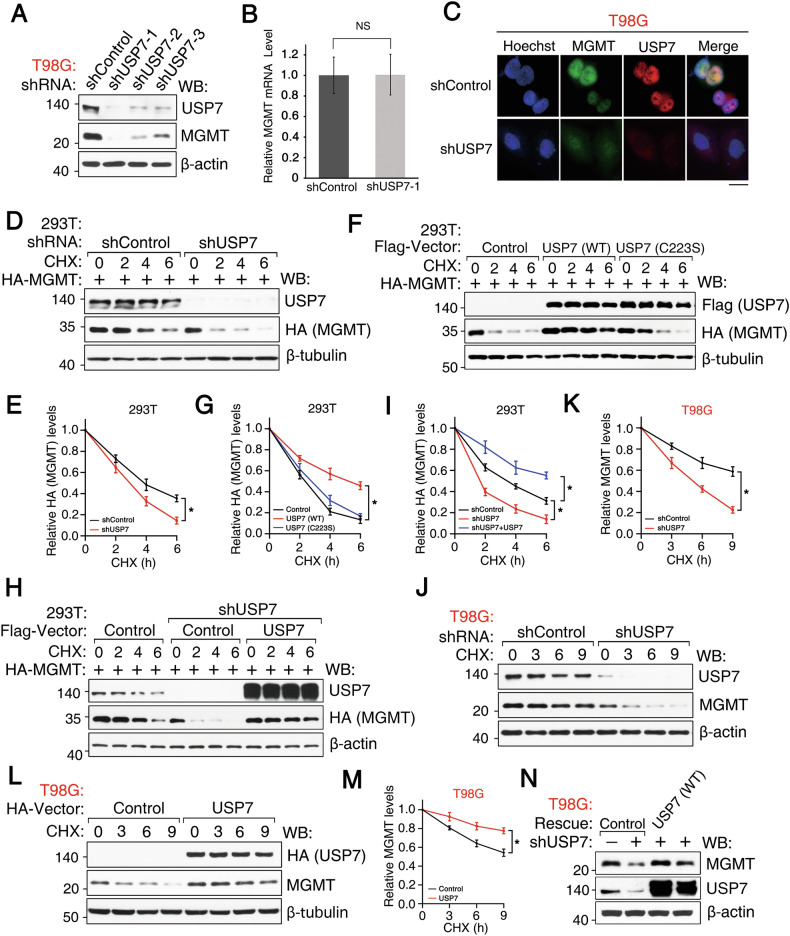
USP7 regulates MGMT stability. **A** Western blotting analysis of MGMT and USP7 levels in T98G cells transduced with control or USP7-specific shRNAs; β-actin loading control. **B** Relative MGMT mRNA levels by qRT-PCR in T98G cells transduced with shControl or shUSP7-1; mean ± SD (n = 3), NS, not significant. **C** Immunofluorescence of MGMT (Green) and USP7 (Red) in T98G cells transduced with shControl or shUSP7; Nuclei, Hoechst (Blue); Scale bar, 20 µm. **D** Western blotting analysis of HA-MGMT stability in HEK293T cells with shControl or shUSP7 after CHX treatment (180 µg/mL); β-tubulin loading control. **E** Quantification of relative HA-MGMT levels from (**D**); Data represent mean ± SD (n = 3 independent experiments). **F** Western blotting analysis of HA-MGMT stability in HEK293T cells transfected with Flag-vector (Control), USP7 WT, or USP7 C223S after CHX treatment (180 µg/mL); β-tubulin loading control. **G** Quantification of relative HA-MGMT levels from (F); Data represent mean ± SD (n = 3 independent experiments). **H** Western blotting analysis of HA-MGMT stability rescue in HEK293T cells (shUSP7 + Flag-vector or Flag-USP7 WT) after CHX treatment (180 µg/mL); β-actin loading control. **I** Quantification of relative HA-MGMT levels from **(H)**; Data represent mean ± SD (n = 3 independent experiments). **J** Western blotting analysis of endogenous MGMT stability in T98G cells with shControl or shUSP7 after CHX treatment (180 µg/mL); β-actin loading control. **K** Quantification of relative MGMT levels from (**J**); Data represent mean ± SD (n = 3 independent experiments). **L** Western blotting analysis of endogenous MGMT stability in T98G cells transduced with vector (Control) or HA-USP7 after CHX treatment (180 µg/mL); β-tubulin loading control. **M** Quantification of relative MGMT levels from (**L**); Data represent mean ± SD (n = 3 independent experiments). **N** Western blotting analysis of endogenous MGMT levels in T98G cells transduced with shControl, or shUSP7 + vector (Rescue Control), or shUSP7 + shRNA-resistant USP7 WT; β-actin loading control.

To investigate whether USP7 stabilizes MGMT, USP7 was knocked down in T98G cells using three independent lentivirus-mediated shRNAs, which resulted in reduced MGMT protein levels (Fig. 3A) without affecting MGMT mRNA levels (Fig. 3B) or subcellular localization (Fig. 3C). Cycloheximide (CHX) chase assays demonstrated that USP7 knockdown shortened the half-life of exogenously expressed HA-MGMT in HEK293T cells (Fig. 3D, E). Conversely, overexpression of wild-type USP7, but not the catalytically inactive C223S mutant, increased MGMT protein levels and extended its half-life compared to cells transfected with empty vector control (Fig. 3F, G). In USP7-depleted cells, USP7 re-expression similarly extended HA-MGMT half-life (Fig. 3H, I). These data indicate that USP7 catalytic activity contributes to post-translational MGMT stabilization.

To assess the physiological relevance, we evaluated endogenous MGMT stability in GBM cells. USP7 knockdown and overexpression, respectively, shortened and prolonged endogenous MGMT half-life in T98G cells (Fig. 3J, K, L, M, respectively). Notably, re-expression of wild-type, but not C223S mutant, USP7 restored MGMT protein levels in USP7-depleted T98G cells (Fig. 3N), linking USP7 catalytic activity to MGMT stabilization in brain tumor cells. Collectively, these results support the role of the catalytic activity of USP7 in regulating MGMT stability in GBM cells.

### Pharmacological inhibition of USP7 impairs alkylation repair by destabilizing MGMT, XPC, ALKBH2, and ALKBH3

**Fig. 4:**
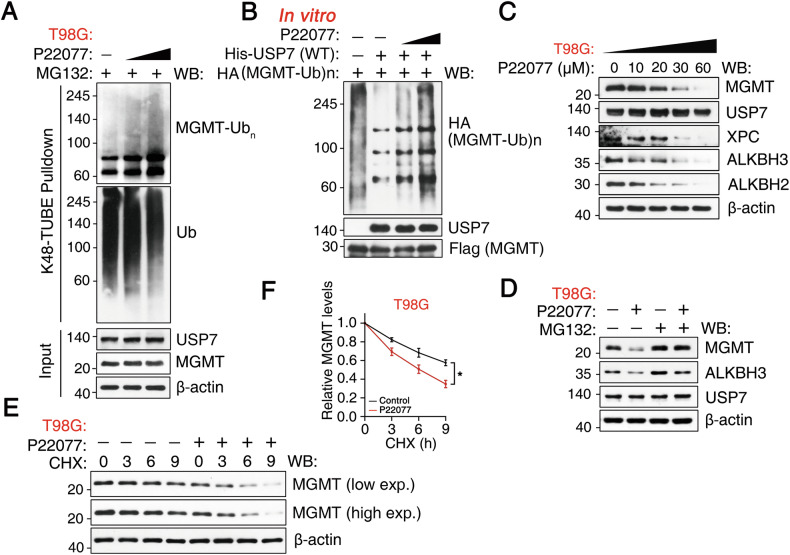
Pharmacological inhibition of USP7 destabilizes MGMT. **A** T98G cells were treated with increasing concentrations of P22077 in the presence of MG132 (20 µM), followed by K48-TUBE pulldown under denatured conditions and western blotting analysis. **B** USP7 deubiquitinates MGMT in vitro. Ubiquitinated MGMT was incubated with purified USP7 in the presence of varying concentrations of P22077 for 4 h, followed by western blotting. **C** Western blotting analysis of MGMT, USP7, XPC, ALKBH3, and ALKBH2 protein levels in T98G cells treated with increasing concentrations of P22077; β-actin loading control. **D** Western blotting analysis of MGMT, ALKBH3, and USP7 protein levels in T98G cells treated with P22077 (10 µM) ± MG132 (20 µM); β-actin loading control. **E** T98G cells were pre-treated with P22077 (30 µM) for 1 h, followed by treatment with CHX (180 µg/mL) for the indicated time, followed by western blotting. **F** Quantification of relative MGMT levels from (**E**); Data represent mean ± SD (n = 3 independent experiments).

Given the role of USP7 in stabilizing MGMT, we hypothesized that pharmacological inhibition of USP7 would promote MGMT degradation. To test this, T98G cells were treated with P22077, a potent USP7 inhibitor. P22077 increased MGMT ubiquitination in a dose-dependent manner (Fig. 4A), which was further confirmed by an in vitro deubiquitination assay (Fig. 4B). Importantly, P22077 triggered dose-dependent degradation of MGMT, as well as other key alkylation repair proteins: XPC (involved in base excision repair), ALKBH2 (involved in demethylation repair), and ALKBH3 (also involved in demethylation repair) (Fig. 4C). This degradation was prevented by the proteasome inhibitor MG132 (Fig. 4D). P22077 also decreased the half-life of endogenous MGMT in T98G cells (Fig. 4E, F), suggesting that MGMT degradation results from protein destabilization. Collectively, these data demonstrate that inhibition of USP7 with P22077 impairs all three major alkylation repair pathways by promoting the proteasomal degradation of key proteins, including MGMT, XPC, ALKBH2, and ALKBH3.

### USP7 counteracts TMZ-induced MGMT ubiquitination and degradation

**Fig. 5:**
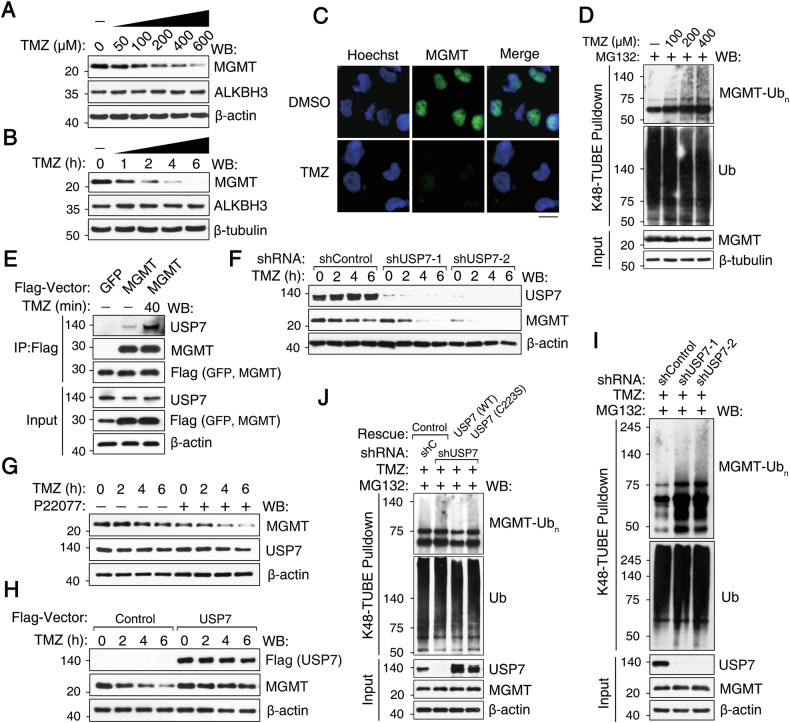
USP7 counteracts TMZ-induced MGMT ubiquitination and degradation. **A** Western blotting analysis of MGMT expression in T98G cells treated with the indicated doses of TMZ for 3 h. **B** Western blotting analysis of MGMT expression in T98G cells treated with 200 µM TMZ for the indicated time periods. **C** T98G cells were treated with 100 µM TMZ for 4 h, followed by immunofluorescence staining using an anti-MGMT antibody, with Hoechst counterstaining for nuclei. Scale bar: 20 µm. **D** T98G cells were treated with the indicated doses of TMZ for 3 h, followed by K48-TUBE pulldown under denatured conditions and western blotting. **E** T98G cells transduced with Flag-tagged GFP or Flag-MGMT were treated with 300 µM TMZ for 40 min, followed by immunoprecipitation and western blotting. **F** T98G cells transduced with control or USP7-specific shRNAs were treated with 100 µM TMZ for the indicated time periods, followed by western blotting. **G** Western blotting analysis of MGMT expression in T98G cells treated with TMZ (100 μM) in the presence or absence of P22077 (10 μM). **H** Western blotting analysis of MGMT expression in T98G cells transduced with Flag-USP7 and treated with TMZ (100 μM) as indicated. **I** T98G cells stably expressing control or two independent USP7-specific shRNAs treated with 100 µM TMZ and 20 µM MG132 for 6 h. **J** T98G cells stably expressing shRNA-resistant Flag-USP7 (WT or catalytically inactive C223S) or empty vector control were transduced with control or USP7-specific shRNA vectors and selected, followed by K48-TUBE pulldown under denatured conditions and western blotting.

Temozolomide (TMZ) is a potent alkylating agent used clinically to treat GBM [2]. To examine the effect of TMZ on MGMT stability, T98G cells were treated with varying concentrations and durations of TMZ and subsequently analyzed by western blotting. TMZ induced MGMT degradation, but not demethylase ALKBH3, in T98G cells in a time- and dose-dependent manner (Fig. 5A, B). MGMT degradation did not correlate with changes in MGMT subcellular localization (Fig. 5C); however, it correlated with a dose-dependent increase in MGMT ubiquitination (Fig. 5D). TMZ treatment increased the interaction between MGMT and USP7 (Fig. 5E), suggesting that increased MGMT interaction with USP7 may be a cellular response to counteract TMZ-induced ubiquitination.

Next, we investigated the role of USP7 in maintaining MGMT stability during TMZ treatment. USP7 knockdown using two distinct shRNAs or inhibition with P22077 enhanced TMZ-induced MGMT degradation in a time-dependent manner (Fig. 5F, G). In contrast, USP7 overexpression stabilized MGMT and attenuated TMZ-induced MGMT degradation (Fig. 5H). To further investigate the role of USP7 in TMZ-induced MGMT ubiquitination, cells were subjected to USP7 knockdown and TMZ treatment, which resulted in increased MGMT ubiquitination, as demonstrated using two independent shRNAs (Fig. 5I). Importantly, re-expression of wild-type USP7 (WT), but not the catalytically inactive C223S mutant, reduced MGMT ubiquitination (Fig. 5J). Taken together, these results demonstrate that USP7 maintains MGMT expression under basal conditions and counteracts TMZ-induced MGMT ubiquitination and degradation during treatment, thus indicating its dual role in GBM TMZ resistance.

### USP7 promotes TMZ resistance by stabilizing MGMT in GBM cells

**Fig. 6:**
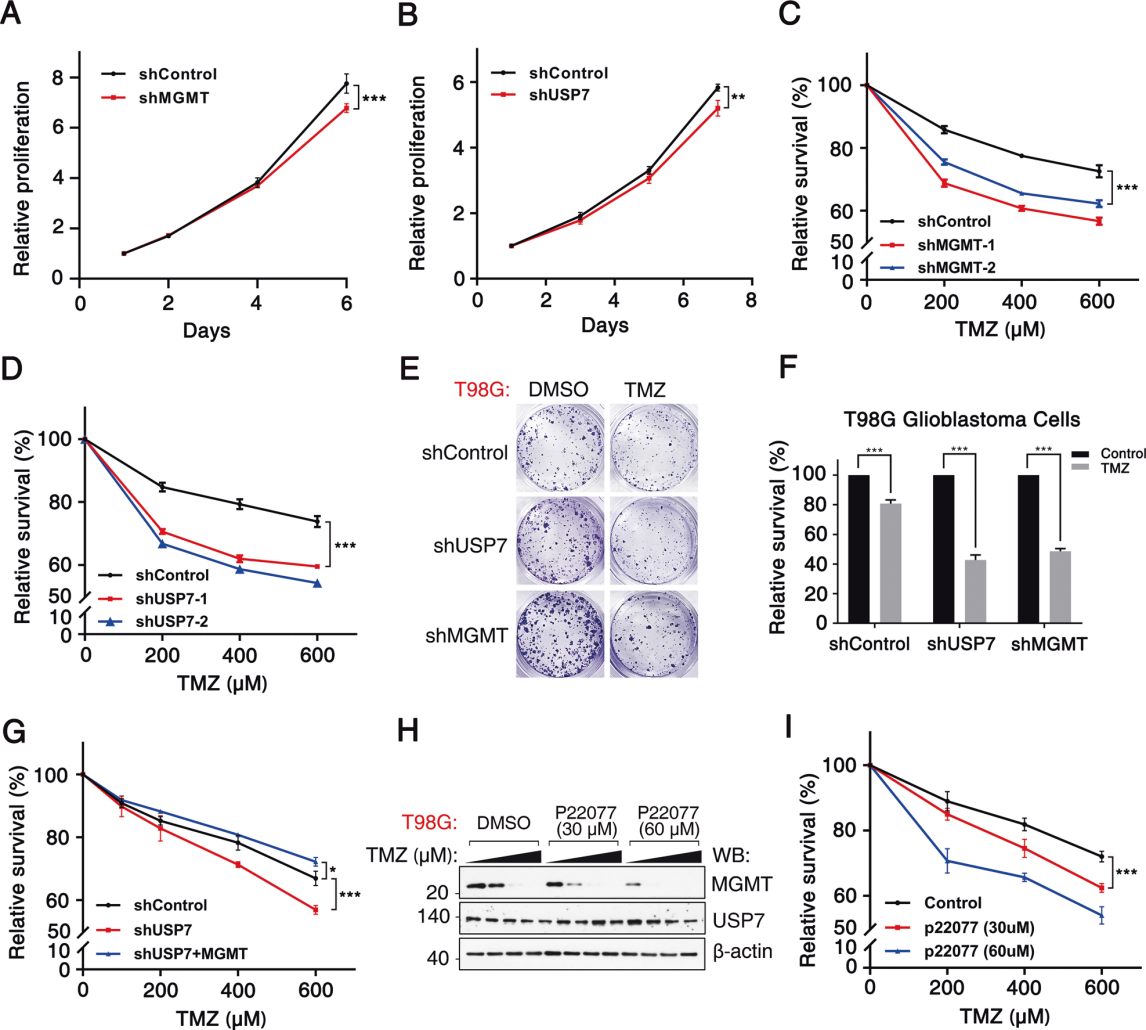
USP7 promotes TMZ resistance by stabilizing MGMT in GBM cells. **A** T98G cells were transduced with MGMT-specific shRNA followed by western blotting analysis to confirm knockdown efficiency. **B** T98G cells were transduced with USP7-specific shRNA, followed by western blotting analysis to confirm knockdown efficiency. **C** T98G cells were transduced with MGMT shRNAs and treated with TMZ at the indicated concentrations for 48 h, and cell viability was assessed relative to untreated control cells using a CCK-8 assay. **D** T98G cells were transduced with USP7-specific shRNAs and treated with TMZ at the indicated concentrations for 48 h, followed by cell viability assessment using a CCK-8 assay. **E** T98G cells transduced with MGMT- or USP7-specific shRNAs were treated with TMZ, followed by colony formation assays after 10 days. **F** Quantification of colony number in panel (**E**). **G** USP7-deficient (generated by shRNA-mediated knockdown) cells transduced with MGMT were treated with TMZ at the indicated concentrations for 48 h, followed by cell viability assessment using a CCK-8 assay. **H** Western blotting analysis of MGMT expression in T98G cells treated with P22077 at the indicated concentrations. **I** The viability of T98G cells co-treated with P22077 and TMZ at the indicated concentrations was assessed using a CCK-8 assay. All data are expressed as the mean ± SD, with at least three biological replicates (n ≥ 3). Statistical significance was analyzed using Student’s t test (*p < 0.05, **p < 0.01, ***p < 0.001).

Given the role of USP7 in stabilizing MGMT, a key factor in DNA alkylation repair and TMZ resistance in GBM [42, 43], we investigated whether USP7 promotes TMZ resistance. Knockdown of MGMT or USP7 in T98G cells reduced cell proliferation (Fig. 6A, B) and significantly increased TMZ sensitivity (Fig. 6C, D). Colony formation assays further confirmed the increased TMZ sensitivity following USP7 or MGMT knockdown (Fig. 6E, F). To determine whether USP7-mediated TMZ resistance depends on MGMT stabilization, we re-expressed MGMT in GBM cells following USP7 knockdown. MGMT re-expression partially restored TMZ resistance in USP7-depleted cells, indicating that USP7 confers chemoresistance at least in part by stabilizing MGMT (Fig. 6G). Additionally, treatment with the USP7 inhibitor P22077 induced dose-dependent MGMT degradation and sensitized GBM cells to TMZ (Fig. 6H, I). Collectively, these data demonstrate that USP7 stabilizes MGMT to promote TMZ resistance, thus suggesting that targeting USP7 may be a potential therapeutic strategy to overcome chemoresistance in GBM.

### Correlation between USP7 and MGMT expression in human GBM

**Fig. 7:**
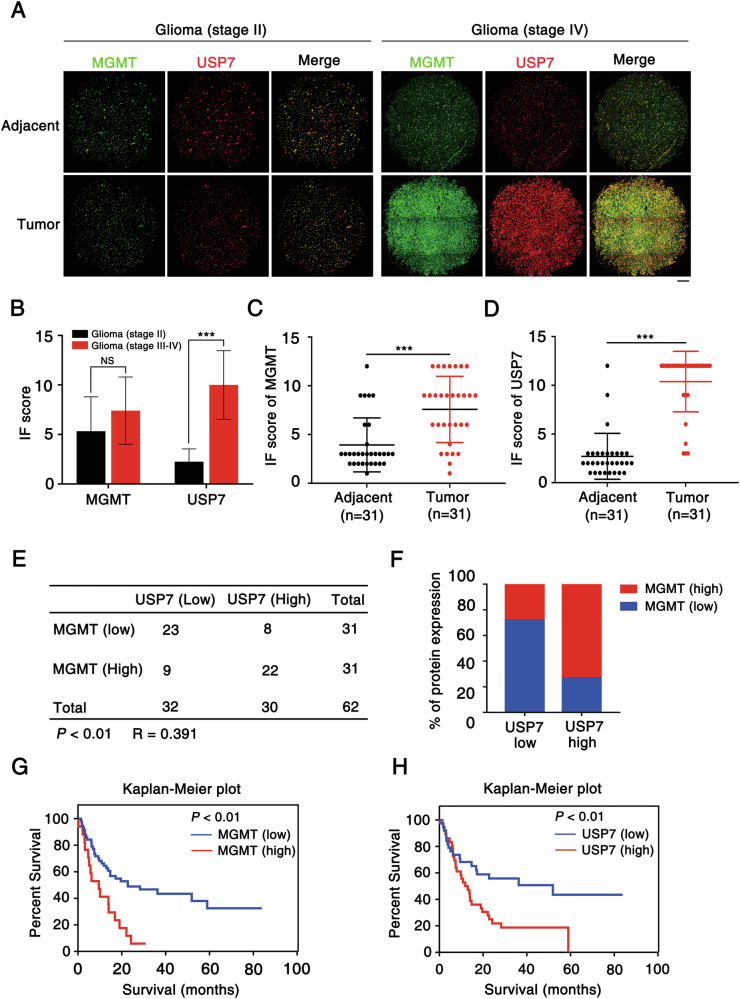
Correlation between USP7 and MGMT expression in human GBM. **A** Representative multi-fluorophore immunofluorescence staining images of a glioblastoma sample on the tissue microarray (TMA). Scale bar: 200 µm. **B** Bar graph showing immunofluorescence (IF) staining scores for MGMT and USP7 in glioma samples of varying grades (n = 12 low-grade, n = 34 high-grade). **C, D** IF staining scores for MGMT or USP7 expression in 31 paired glioblastoma samples. Statistical analysis for panels **B-D** was performed using Student’s t test (***p < 0.001). **E** Correlation analysis of USP7 and MGMT expression in human glioblastoma tissue samples. Statistical analysis was performed using the Pearson χ² test, with *R* representing the Pearson correlation coefficient. **F** Quantification of the data in panel **E** revealed a significant correlation (**p < 0.01) between USP7 and MGMT expression. **G**, **H** Overall survival curves were generated using the PrognoScan database based on USP7 and MGMT expression levels in patients with glioma.

We performed immunofluorescence (IF) staining on a glioma tissue microarray (TMA) comprising 92 glioma samples and adjacent non-tumor tissues to assess the correlation between USP7 and MGMT expression (Fig. 7A). USP7 expression was significantly lower in low-grade gliomas (grade II) compared with high-grade gliomas (grades III and IV) (Fig. 7B; *p* < 0.01). MGMT expression was positively correlated with USP7 levels in GBM (grade IV) tissues, while both markers exhibited low expression in adjacent normal tissues (Fig. 7C, D; *p* < 0.01, n = 31 paired glioblastoma samples). Quantitative analysis of IF staining revealed a positive correlation between USP7 and MGMT expression levels (Fig. 7E, F; *p* < 0.01, n = 62 glioblastoma samples).

The prognostic significance of USP7 and MGMT in gliomas was assessed using the PrognoScan database [44]. GBM patients with elevated USP7 expression exhibited a poorer prognosis, similar to those with elevated MGMT expression (Fig. 7G, H). Although the association of MGMT with glioma prognosis is well-established [17, 45], our results indicate that USP7 may serve as both a GBM prognostic marker and a therapeutic target, potentially functioning in association with MGMT, as suggested by our TMA IF data.

## Discussion

Previously, we demonstrated that USP7 indirectly promotes DNA demethylation, a key mechanism in the repair of alkylation damage, by stabilizing demethylases through its interaction with OTUD4, which serves as a substrate scaffold [37]. In this study, we identify a new and direct role of USP7 in MGMT-mediated direct reversal repair, another key alkylation repair mechanism. Using LC-MS/MS analysis, we identified USP7, a DUB known to preferentially cleave K48-linked ubiquitin chains, as a nuclear interactor of MGMT. USP7 directly binds to and colocalizes with MGMT in the nucleus (Fig. 1), counteracting K48-linked ubiquitination and thereby preventing its proteasomal degradation (Figs. 2, 3). These data identify USP7 as the first nuclear DUB shown to stabilize MGMT in vivo. Furthermore, in T98G cells, which are known to rely on MGMT for TMZ resistance, ectopic MGMT overexpression restores alkylation damage repair and reduces sensitivity to TMZ (Fig. 6).

K48-linked ubiquitination, a modification typically signaling proteins for proteasomal degradation [46], is identified here for the first time as a mechanism for MGMT turnover (Fig. 2B, C). While MGMT is known to be localized in the nucleus, where nuclear E3 ligases such as RAD18 and E6AP mediate its degradation [15, 16], our data reveal that USP7 antagonizes this K48-linked ubiquitination, thereby stabilizing MGMT, promoting DNA repair, and counteracting the degradative effects of these nuclear E3 ligases. Cytoplasmic regulators such as TRIM72 [19, 47] and USP19 [17, 20, 21] appear to influence specific subsets of MGMT molecules, potentially through the nuclear-cytoplasmic transport mechanism of MGMT [8]. However, USP7 acts directly within the nucleus to regulate MGMT stability. Taken together, these data identify USP7 as a key regulator of MGMT stability in the nucleus of glioblastoma (GBM) cells by counteracting K48-linked ubiquitination, offering mechanistic insights into MGMT regulation.

Extending our previous work demonstrating USP7’s indirect role in regulating the demethylation repair pathway [37], we now demonstrate that USP7 directly regulates the direct reversal repair pathway by stabilizing MGMT in T98G cells. This observation, combined with its established role in modulating the base excision repair (BER) pathway through CSB and XPC stabilization in HeLa cells [33–36] and our current data showing similar modulation of XPC in GBM cells (Fig. 4C), indicates that USP7 coordinates the activity of all three major alkylation repair pathways: direct reversal, demethylation, and BER. Moreover, our study presents evidence that USP7 stabilizes the demethylation repair proteins ALKBH2 and ALKBH3 in GBM cells (Fig. 4C), expanding upon previous observations in prostate cancer cells [37]. Collectively, these results underscore a central role for USP7 in modulating multiple alkylation repair pathways across different cancer types. Significantly, our data demonstrate that pharmacological inhibition of USP7 with P22077 induces the degradation of MGMT, XPC, ALKBH2, and ALKBH3, consequently impairing all three major alkylation repair pathways in GBM cells. Considering the well-established role of alkylation repair in chemoresistance [7], inhibition of USP7 represents a highly attractive therapeutic strategy to overcome chemoresistance in GBM and other malignancies, potentially facilitating the use of combination therapies with various alkylating agents, including TMZ. In addition to its role in alkylation repair, USP7 contributes to broader DNA damage response pathways, including those involving p53 and Chk1 stabilization [26–31], suggesting a more significant impact on chemotherapeutic response.

USP7 promotes TMZ resistance through two distinct mechanisms. Under basal conditions, USP7 stabilizes MGMT, maintaining its levels and contributing to GBM cell-intrinsic resistance against endogenous alkylation damage (Figs. 3, 6). During TMZ treatment, USP7 antagonizes TMZ-induced MGMT degradation, further maintaining MGMT levels and reducing TMZ-induced cytotoxicity (Figs. 5, 6). These dual mechanisms collectively stabilize MGMT under both basal and TMZ-induced stress conditions, thereby enhancing TMZ resistance in GBM cells. Consistent with this, USP7 knockdown combined with TMZ treatment significantly impaired colony formation (Fig. 6E), supporting the functional role of these dual mechanisms in GBM.

Targeting the USP7-MGMT axis represents a promising strategy for improving treatment efficacy in GBM. P22077, a USP7-specific inhibitor, promotes MGMT degradation and sensitizes GBM cells to TMZ (Figs. 4, 6F), demonstrating the therapeutic potential of this strategy. Beyond GBM, P22077 demonstrates anti-tumor activity in various cancers. In hepatocellular carcinoma (HCC) cells, P22077 induces apoptosis and inhibits cell proliferation, while in neuroblastoma, it promotes p53-mediated apoptosis [24, 48]. P22077 also suppresses melanoma growth and metastasis in vitro and in vivo [25]. However, P22077 and other USP7 inhibitors, such as P5091 and P50429, suffer from dose-dependent cytotoxicity and limited pharmacokinetic profiles [49–54]. These limitations point out the need for developing more selective strategies. As demonstrated with the USP7-MDM2 axis, selective inhibition using HBX 41108 inhibits MDM2 deubiquitination without affecting the USP7-p53 axis, resulting in selective p53 stabilization and MDM2 degradation [55]. This selective inhibition avoids affecting other critical USP7 substrates and minimizes potential side effects. Inspired by this concept, a similar strategy could be developed to selectively target the USP7-MGMT axis. By selectively targeting the USP7-MGMT interaction rather than globally inhibiting USP7, this strategy could enhance MGMT degradation, sensitize GBM cells to TMZ, and reduce the cytotoxicity associated with nonselective USP7 inhibition.

A tissue microarray (TMA) of 92 glioma specimens was used to assess the clinical significance of the USP7-MGMT axis. USP7 was significantly overexpressed in GBM compared with normal brain tissue and strongly correlated with high-level MGMT expression (Fig. 7A–F; *p* < 0.05). High expression levels of USP7 and MGMT were associated with a significantly worse prognosis in GBM patients by Kaplan-Meier analyses (Fig. 7G, H; *p* < 0.01). These data reveal a novel mechanistic connection between the USP7-MGMT axis and GBM progression. While the absence of additional in vivo validation represents a limitation, this study builds upon strong foundational evidence. Depletion of USP7 has previously been shown to inhibit tumor progression in various cancers in murine models [50, 56], and MGMT knockdown in GBM cell-based models reinforces the role of MGMT in TMZ resistance [57]. These established models, coupled with ethical considerations such as minimizing animal use and adhering to the principles of the 3Rs (replacement, reduction, and refinement) [58], mitigate the immediate need for additional animal studies at this stage. Future studies will further develop in vivo models to address unresolved mechanistic questions and explore the therapeutic potential of targeting the USP7-MGMT axis, particularly in overcoming TMZ resistance and enhancing the efficacy of combination therapies with TMZ and other alkylating agents targeting distinct alkylation repair pathways.

## Supplementary information

Table S1

Uncropped WB files for USP7 and MGMT

## Data Availability

All presented data are included in this article and the supplementary file.
