# Supplementary material for: USP7 promotes temozolomide resistance by stabilizing MGMT in glioblastoma
Source: Cell Death Dis. 2025 Aug 20;16(1):631. doi: 10.1038/s41419-025-07969-3 (PMC12368267; doi:10.1038/s41419-025-07969-3)

To Fig. 1

Fig. 1A

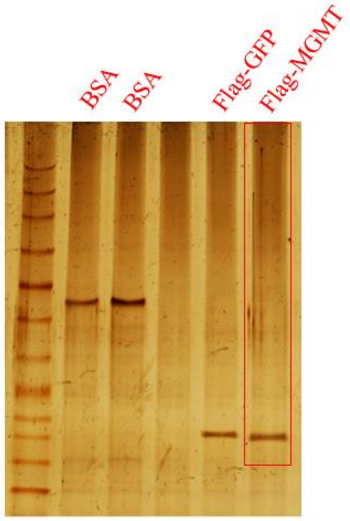

Fig. 1B

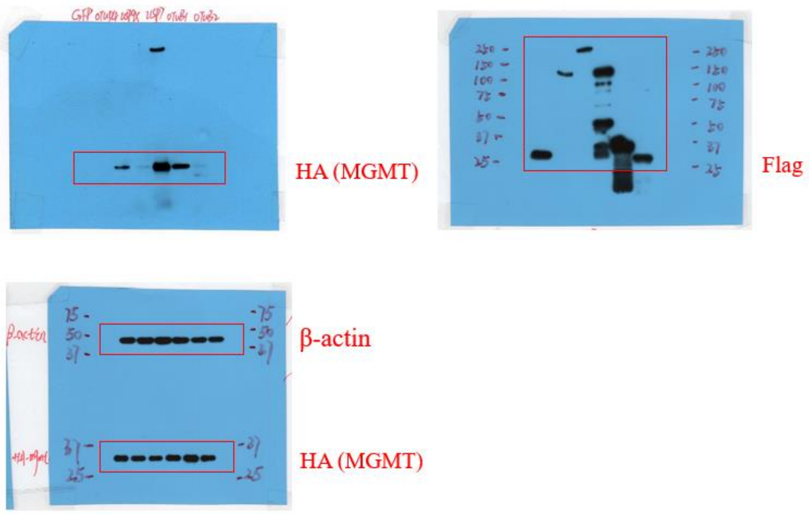

Fig. 1C

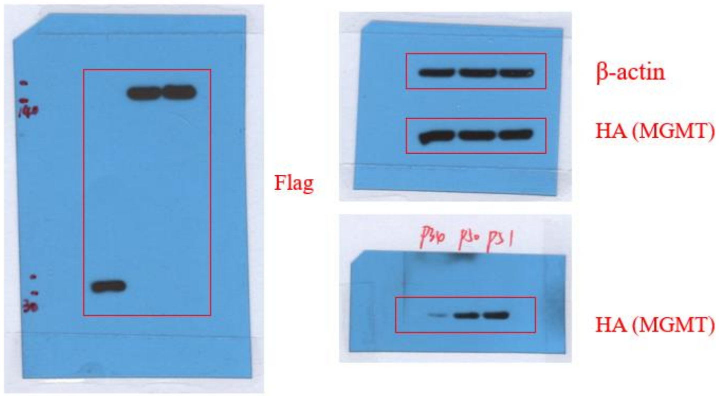

Fig. 1D

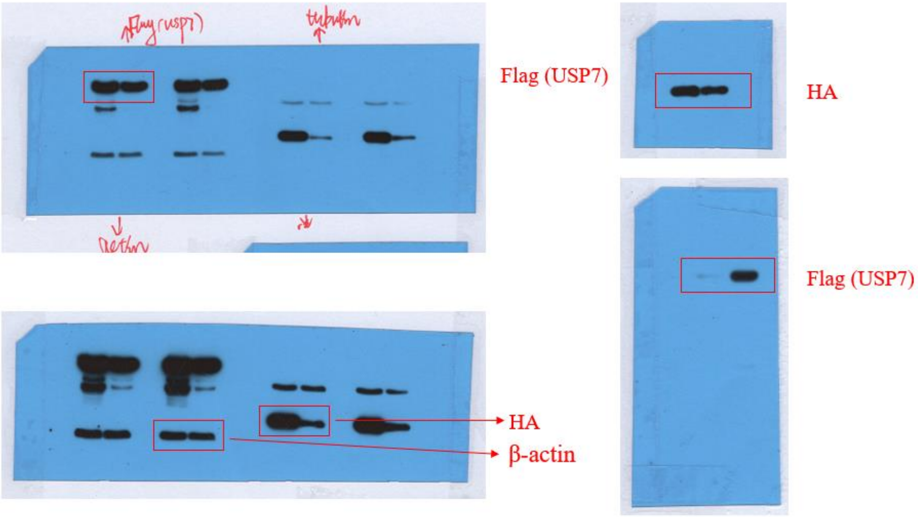

Fig. 1F

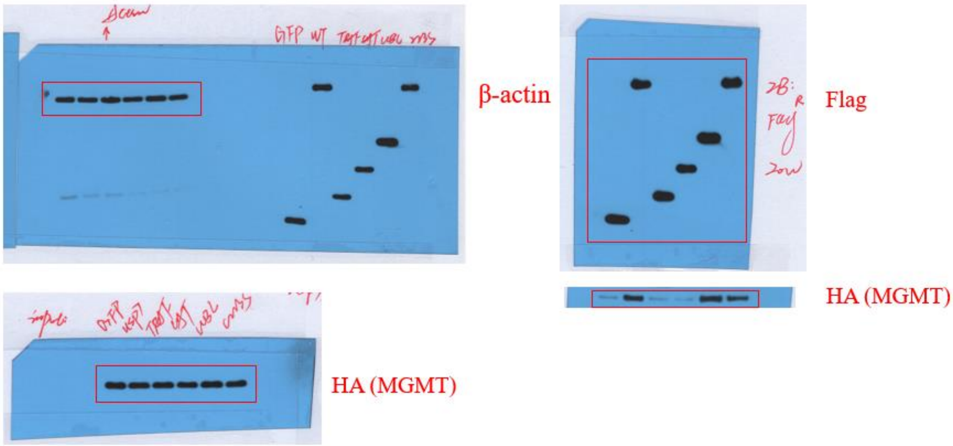

Fig. 1H

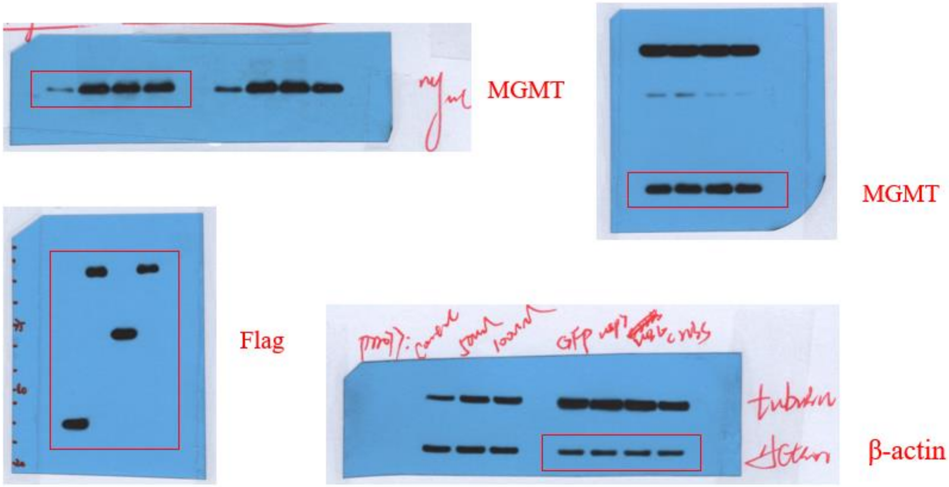

Fig. 1I

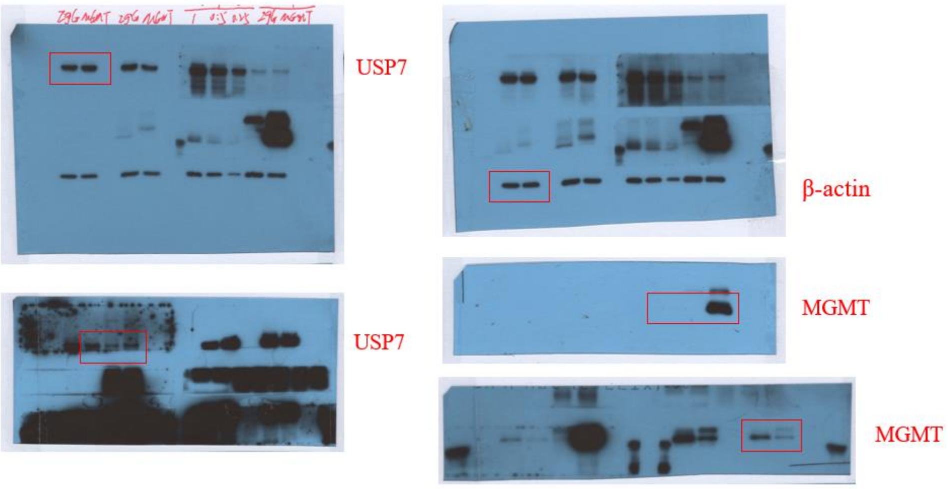

Fig. 1J

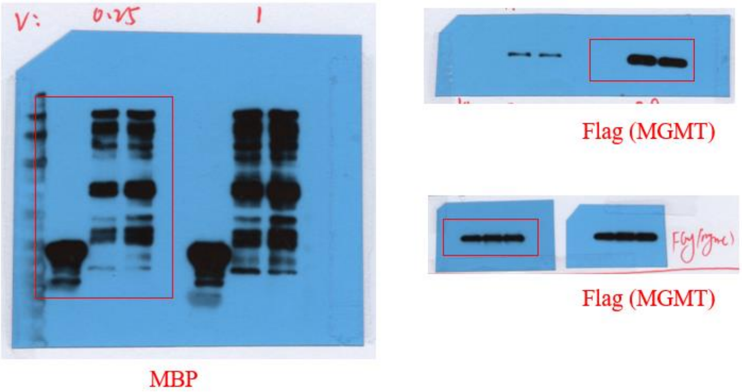

Fig. 1K

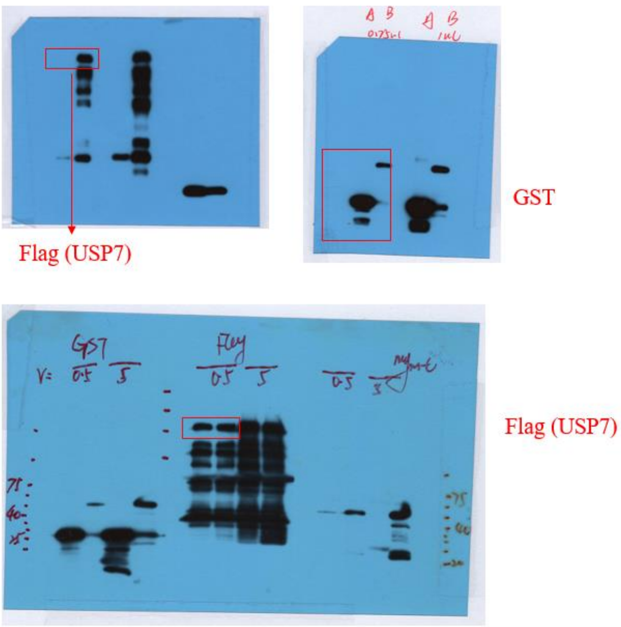

To Fig. 2

Fig. 2A

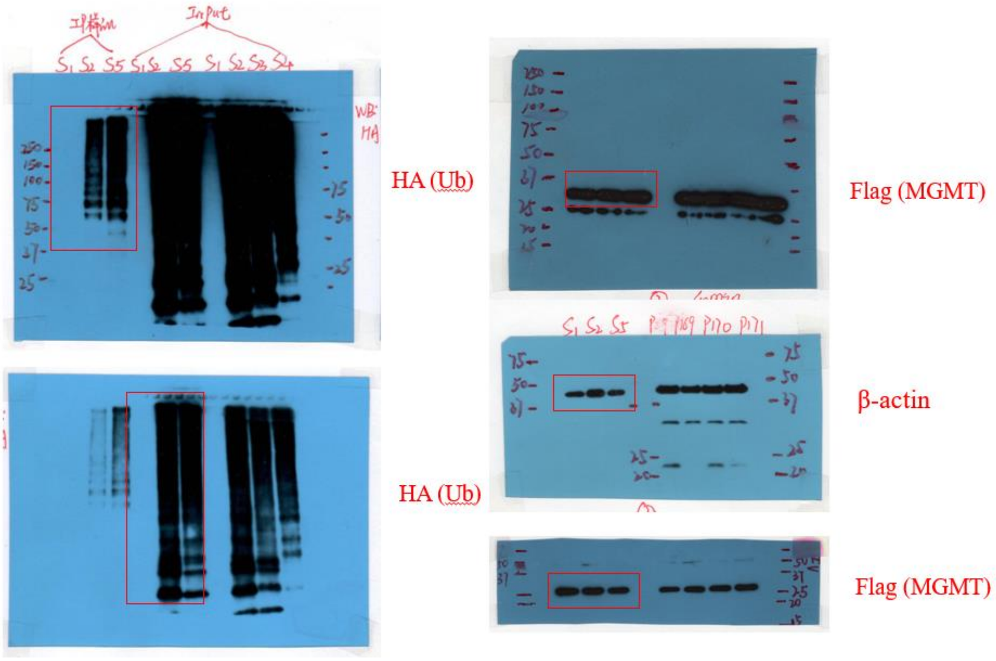

Fig. 2B

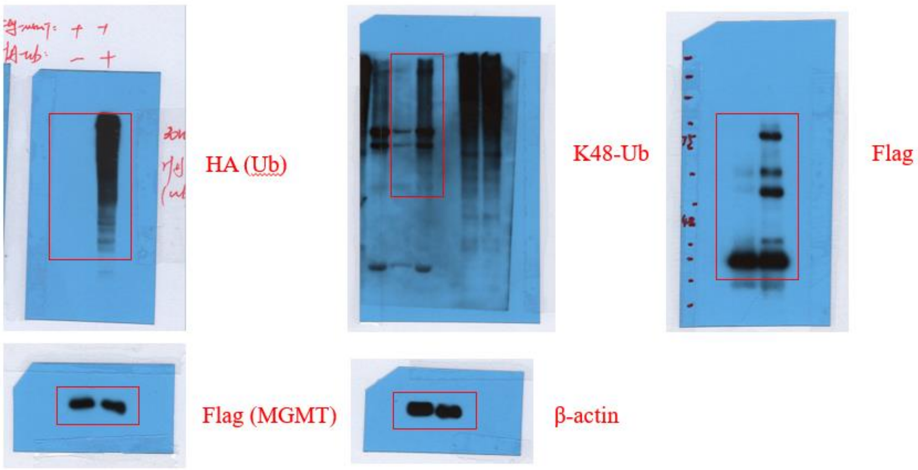

Fig. 2C

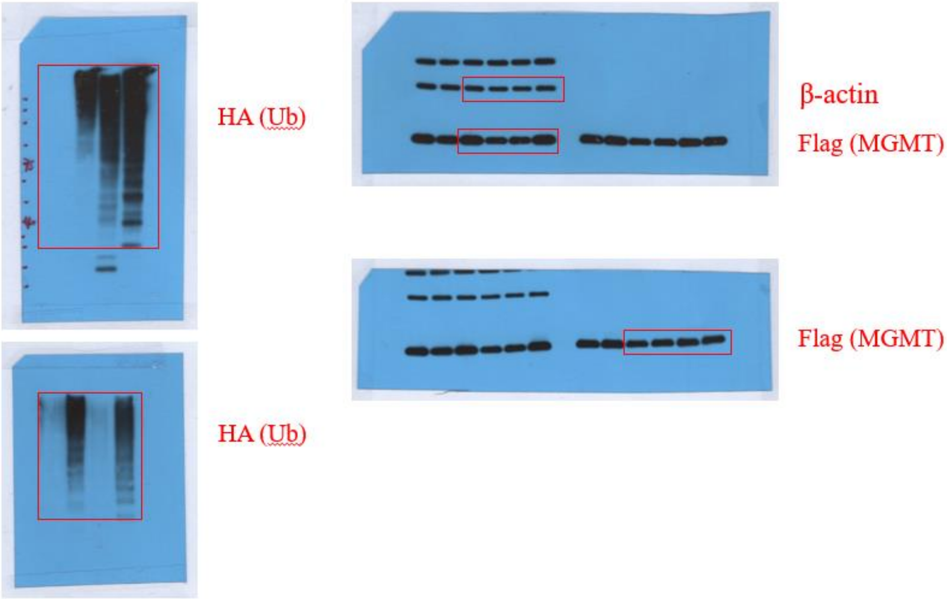
HA (Ub)
HA (Ub)
β-actin
Flag (MGMT)
Flag (MGMT)

Fig. 2D

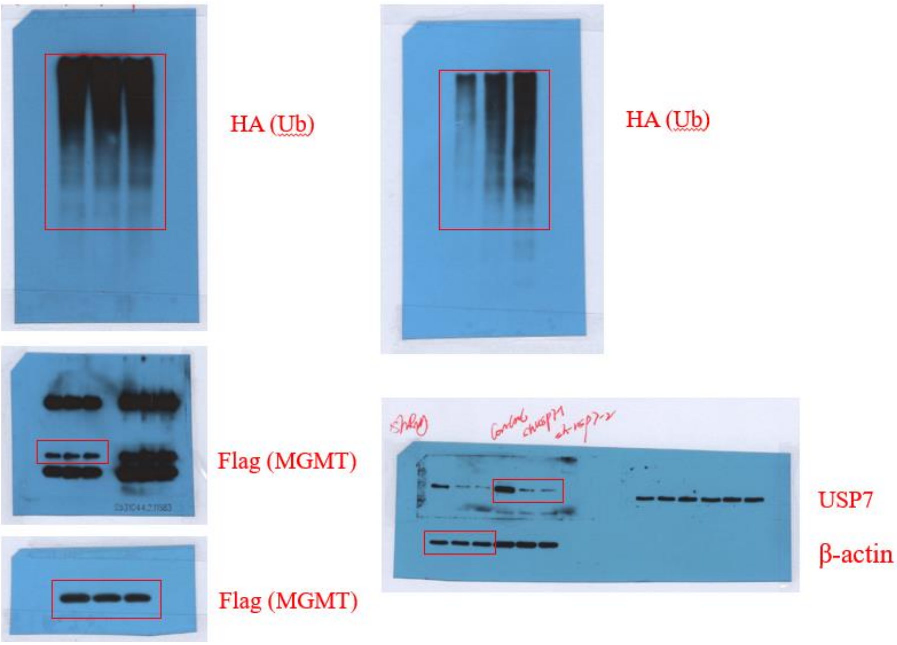
HA (Ub)
HA (Ub)
Flag (MGMT)
Flag (MGMT)
USP7
β-actin

Fig. 2E

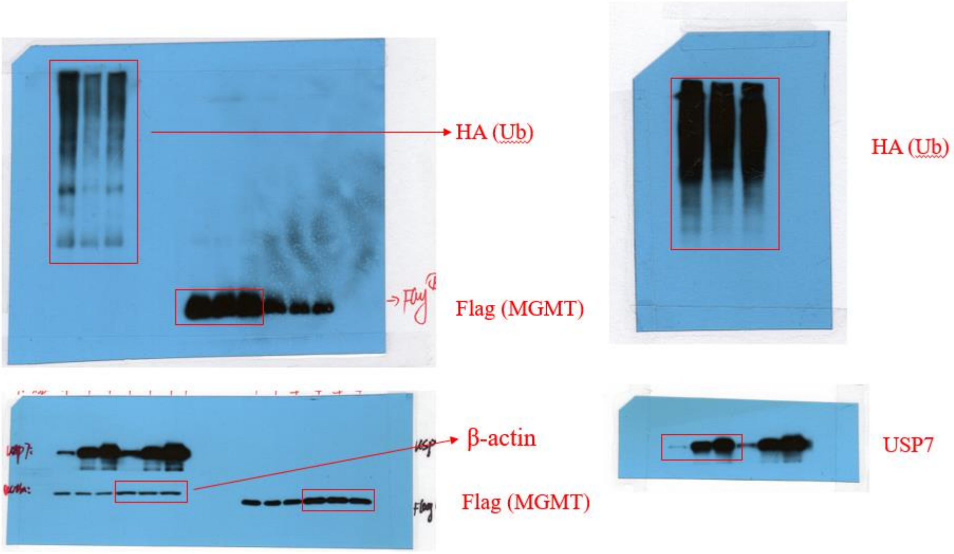
HA (Ub)
Flag (MGMT)
HA (Ub)
β-actin
Flag (MGMT)
USP7

Fig. 2F

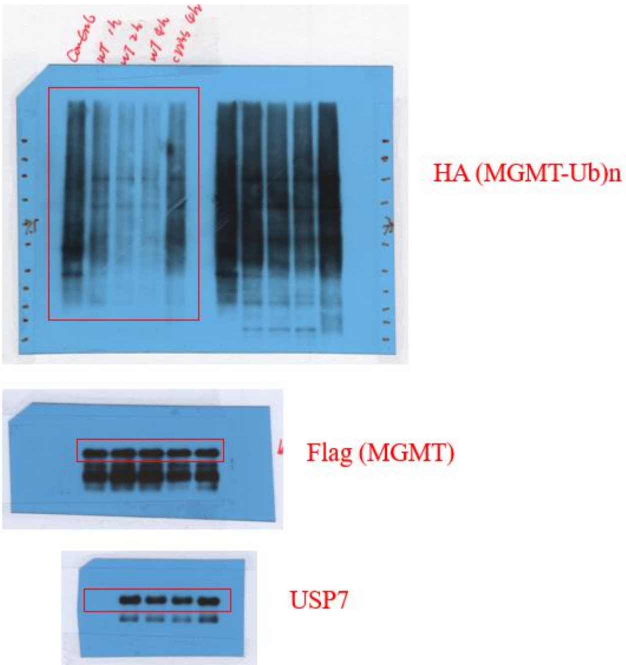
HA (MGMT-Ub)n
Flag (MGMT)
USP7

To Fig. 3

Fig. 3A

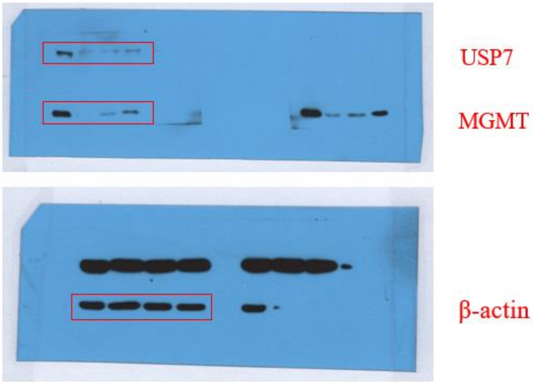

Fig. 3D

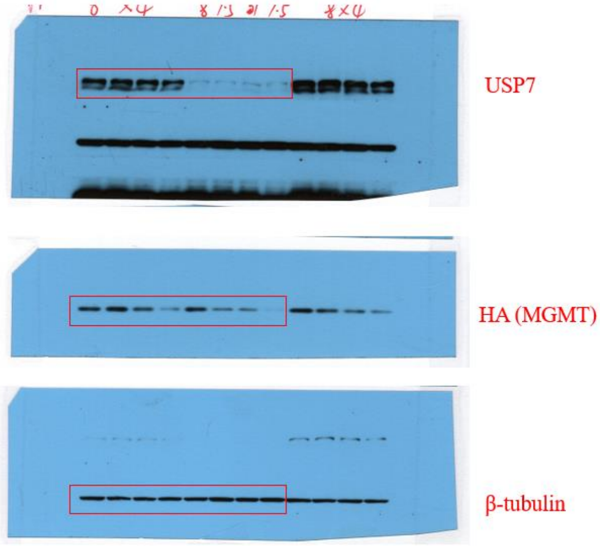

Fig. 3E

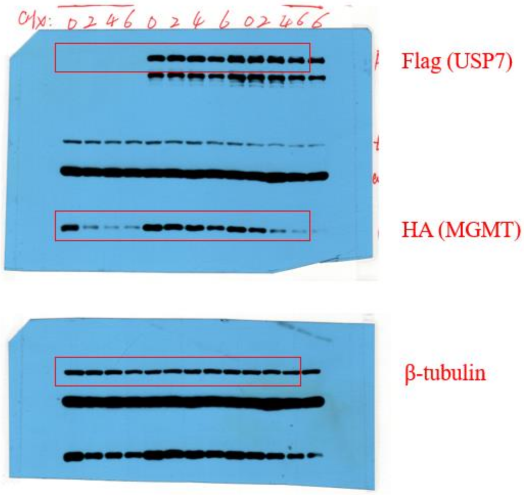

Fig. 3F

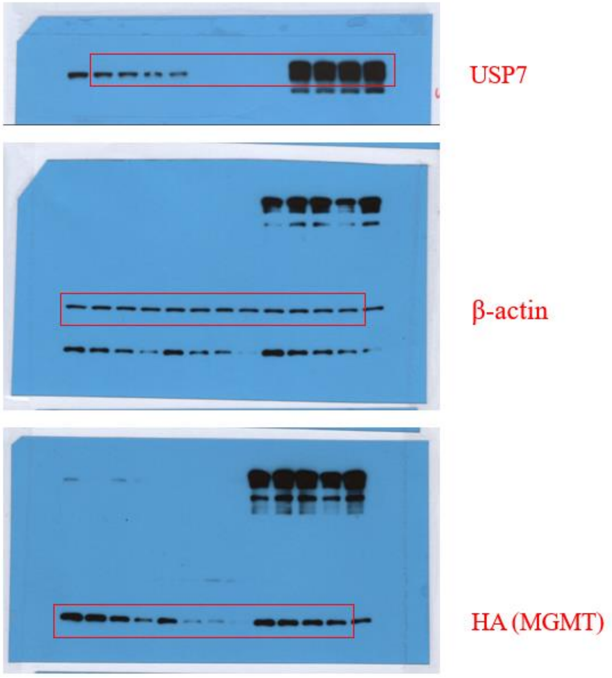

Fig. 3G

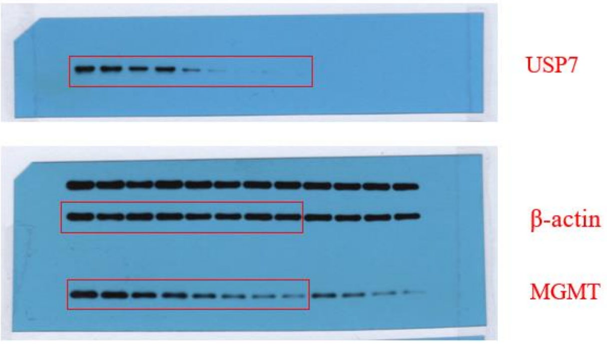

Fig. 3H

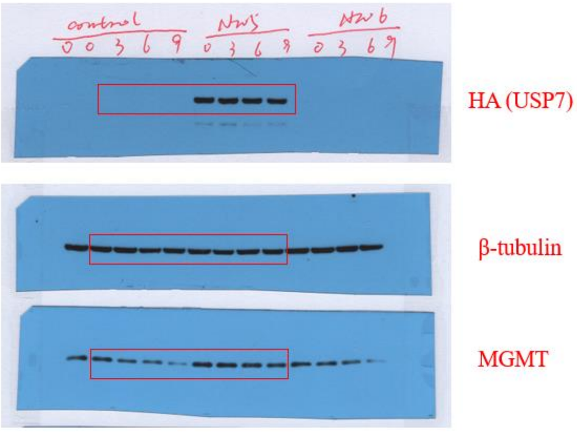

Fig. 3I

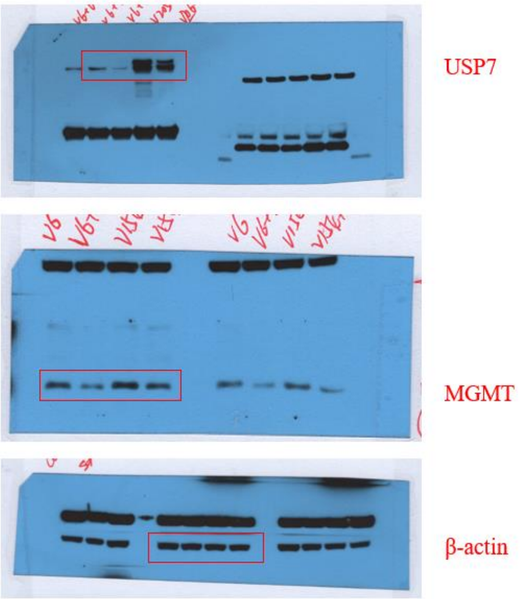

To Fig. 4

Fig. 4A

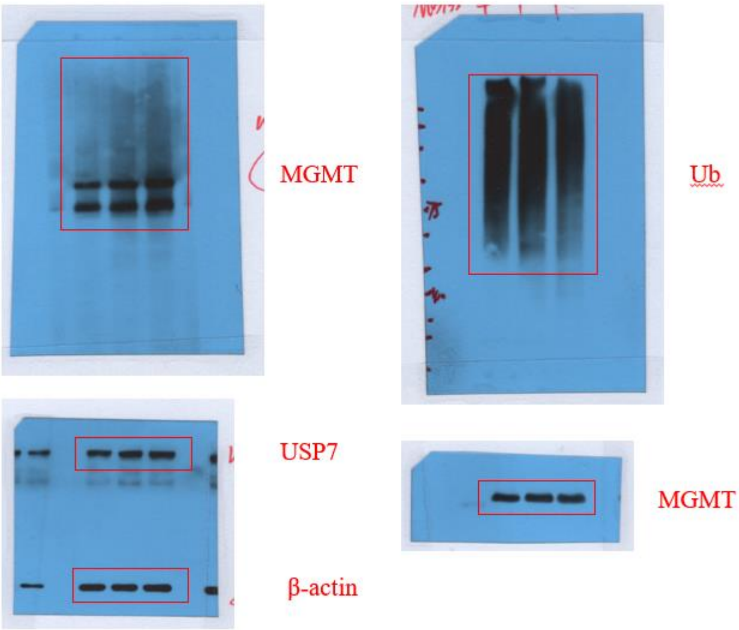

Fig. 4B

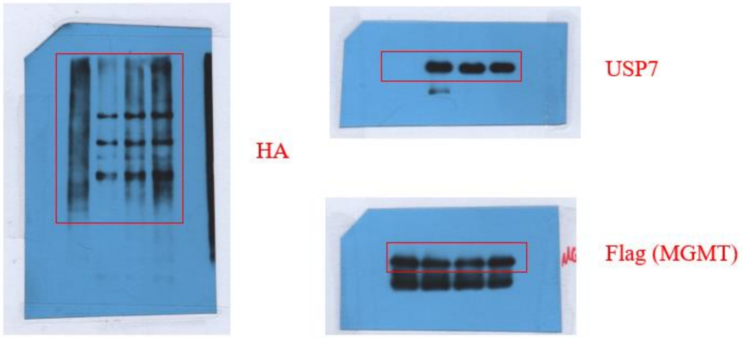

Fig. 4C

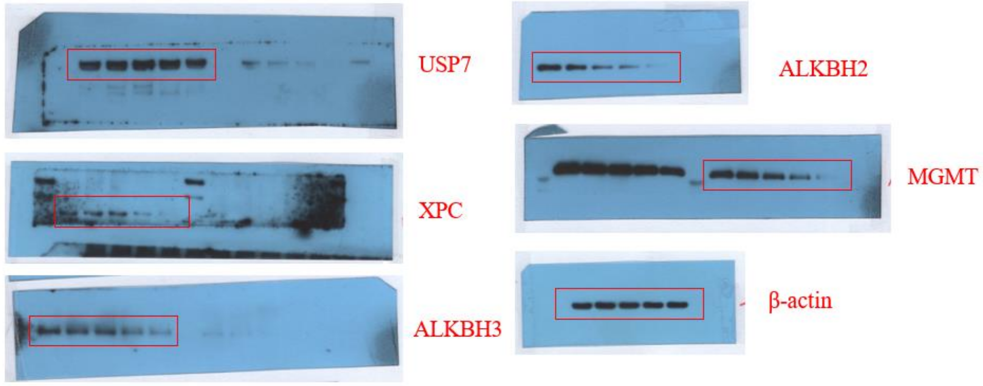

Fig. 4D

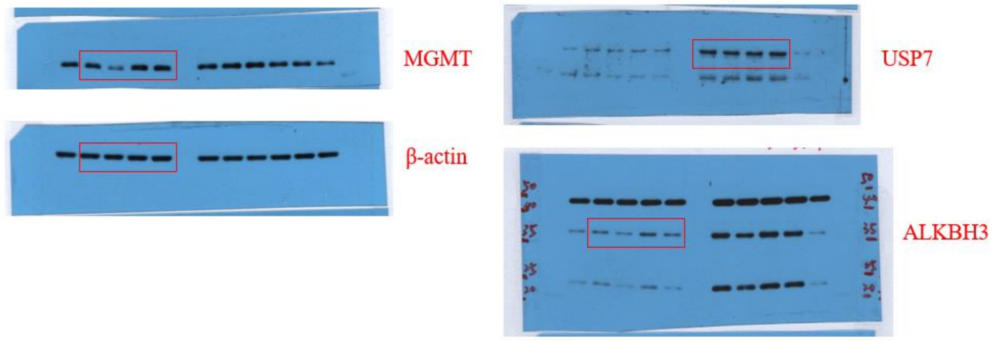

Fig. 4E

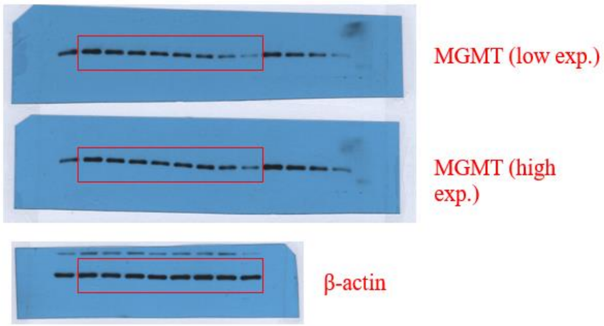

To Fig. 5

Fig. 5A

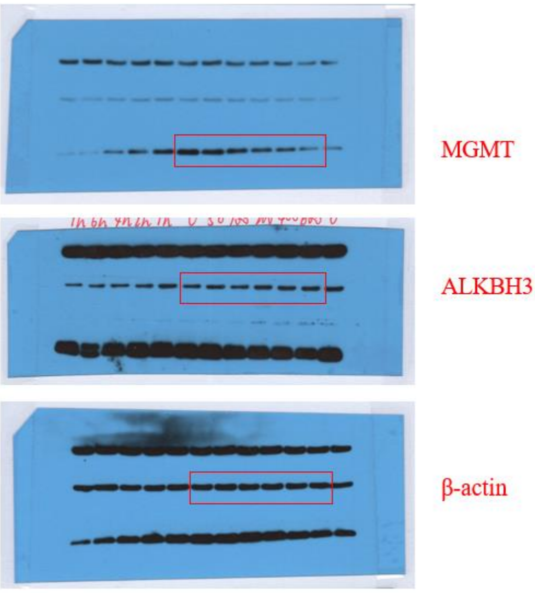

Fig. 5B

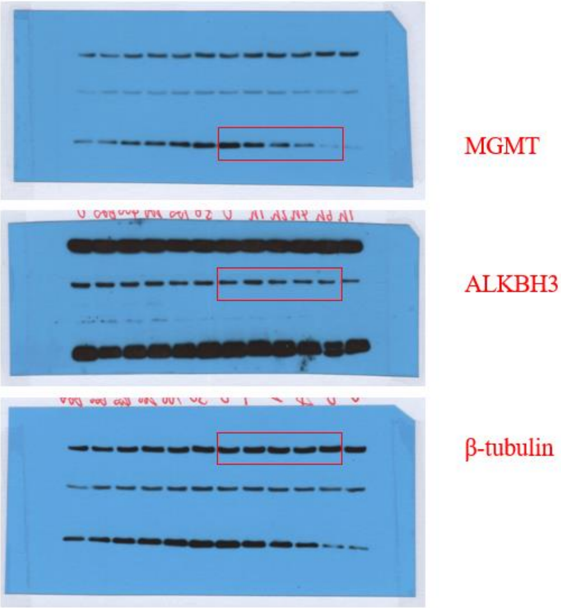

Fig. 5D

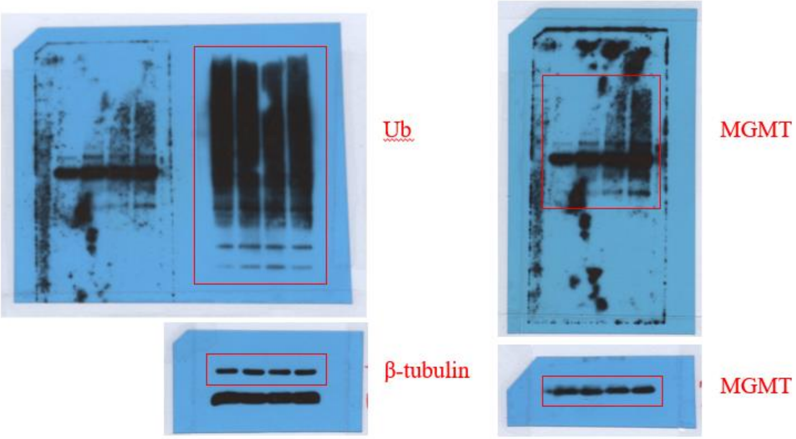

Fig. 5E

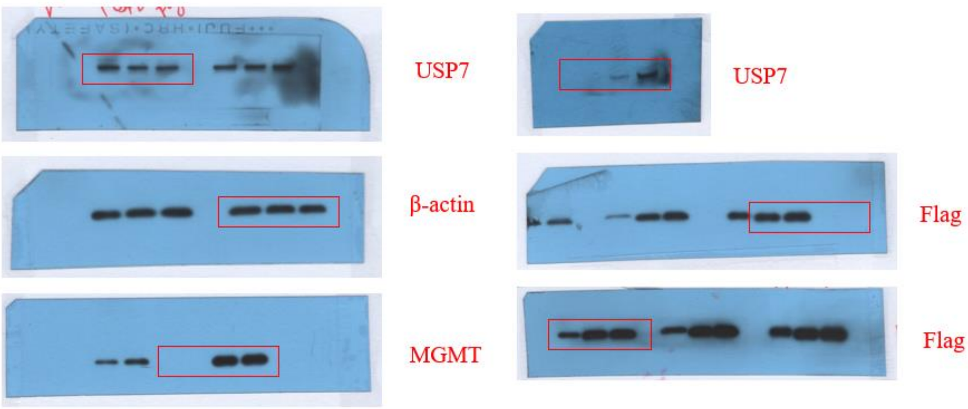

Fig. 5F

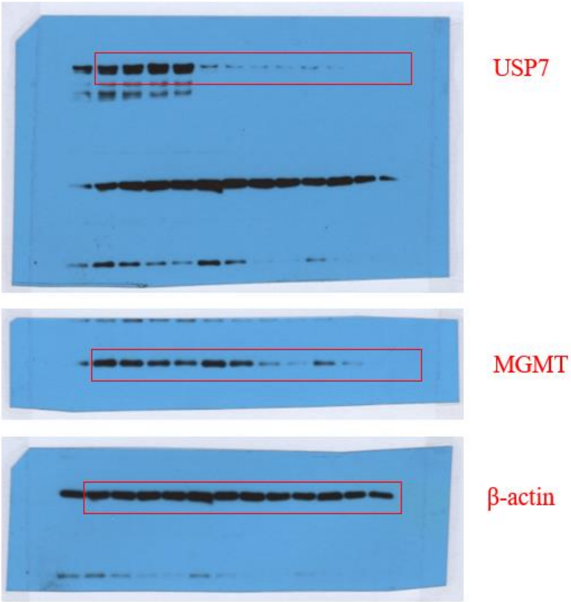

Fig. 5G

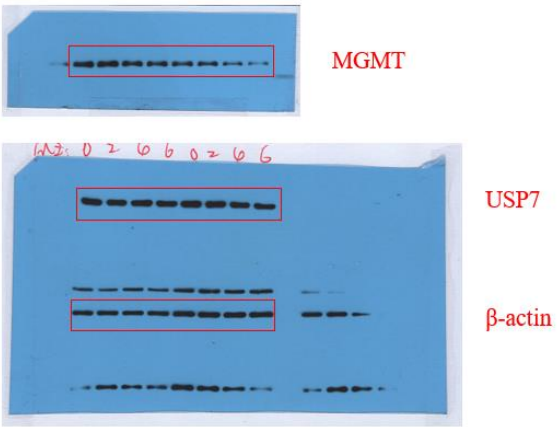

Fig. 5H

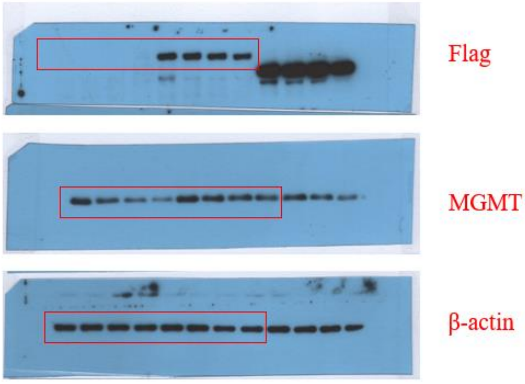

Fig. 5I

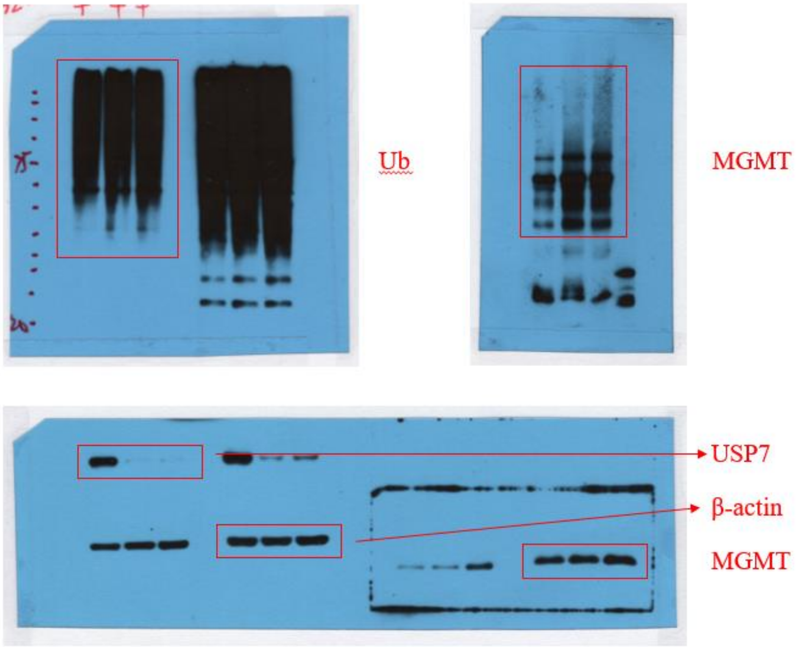

Fig. 5J

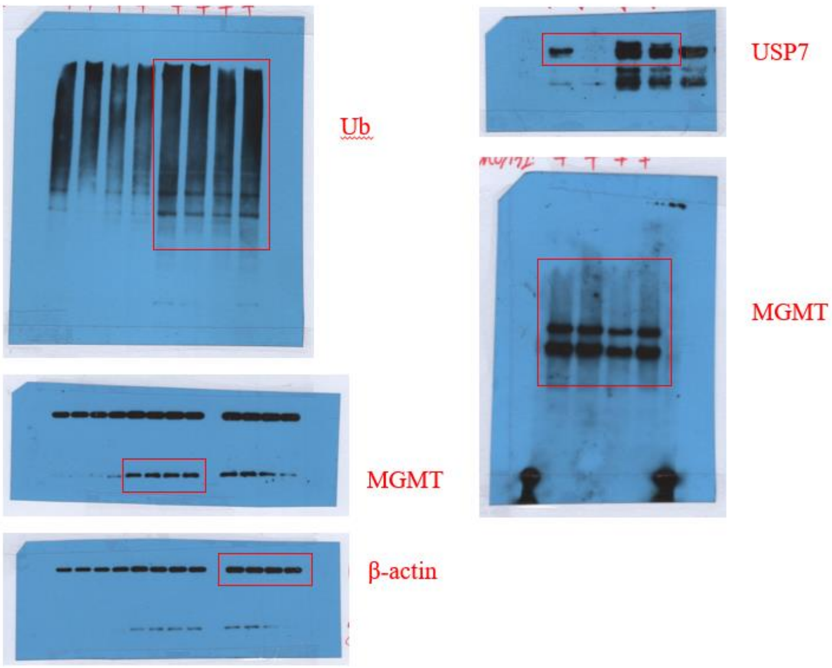

To Fig. 6

Fig. 6H

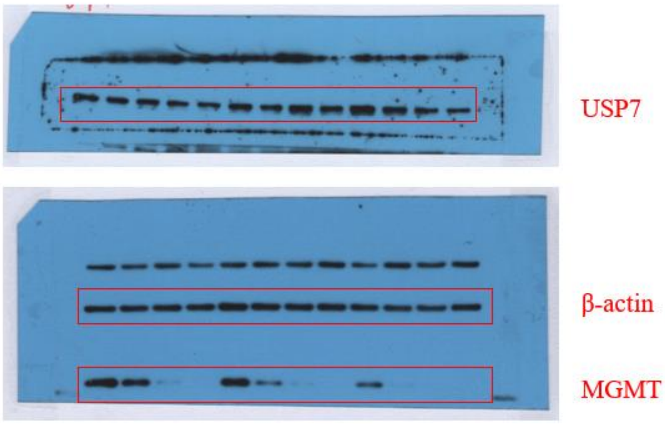

Supplement: Supplementary file 2 — Uncropped WB files for USP7 and MGMT [file 41419_2025_7969_MOESM2_ESM.pdf]
